# Supplementary material for: Matching study using health and police datasets for characterising interpersonal violence in the community of Khayelitsha, South Africa 2013–2015
Source: BMJ Open. 2022 Sep 30;12(9):e048129. doi: 10.1136/bmjopen-2020-048129 (PMC9528606; doi:10.1136/bmjopen-2020-048129)
Supplement: Supplementary data [file bmjopen-2020-048129supp001.pdf]

Supplemental Table 1. Matching proportion per day of week of presentation to the clinical facility

DAY	MATCHED	TOTAL	PERCENT (%)
Monday	14	89	15,7
Tuesday	12	47	25,5
Wednesday	10	44	22,7
Thursday	5	37	13,5
Friday	15	70	21,4
Saturday	27	182	14,8
Sunday	21	239	8,8
