# Supplementary material for: Matching study using health and police datasets for characterising interpersonal violence in the community of Khayelitsha, South Africa 2013–2015
Source: BMJ Open. 2022 Sep 30;12(9):e048129. doi: 10.1136/bmjopen-2020-048129 (PMC9528606; doi:10.1136/bmjopen-2020-048129)
Supplement: Author's manuscript [file bmjopen-2020-048129.draft_revisions.pdf]

BMJ Open is committed to open peer review. As part of this commitment we make the peer review history of every article we publish publicly available.

When an article is published we post the peer reviewers' comments and the authors' responses online. We also post the versions of the paper that were used during peer review. These are the versions that the peer review comments apply to.

The versions of the paper that follow are the versions that were submitted during the peer review process. They are not the versions of record or the final published versions. They should not be cited or distributed as the published version of this manuscript.

BMJ Open is an open access journal and the full, final, typeset and author-corrected version of record of the manuscript is available on our site with no access controls, subscription charges or pay-per-view fees (http://bmjopen.bmj.com).

If you have any questions on BMJ Open's open peer review process please email info.bmjopen@bmj.com

BMJ Open

Merging Health and Police datasets for characterising interpersonal violence in the community of Khayelitsha, South Africa 2013-2015

Journal:	*BMJ Open*
Manuscript ID	bmjopen-2020-048129
Article Type:	Original research
Date Submitted by the Author:	19-Dec-2020

Complete List of Authors:	Jabar, Ardil; University of Cape Town, School of Public Health and Family Medicine<br>Oni, Tolu; University of Cape Town, School of Public Health and Family Medicine; University of Cambridge, MRC Epidemiology Unit<br>London, Leslie; University of Cape Town, School of Public Health and Family Medicine<br>Cois, Annibale ; University of Cape Town Faculty of Health Sciences, Division of Epidemiology and Biostatistics<br>Matzopoulos, Richard; University of Cape Town, School of Public Health and Family Medicine

Keywords:	PUBLIC HEALTH, ACCIDENT & EMERGENCY MEDICINE, EPIDEMIOLOGY

SCHOLARONE™
Manuscripts

I, the Submitting Author has the right to grant and does grant on behalf of all authors of the Work (as defined in the below author licence), an exclusive licence and/or a non-exclusive licence for contributions from authors who are: i) UK Crown employees; ii) where BMJ has agreed a CC-BY licence shall apply, and/or iii) in accordance with the terms applicable for US Federal Government officers or employees acting as part of their official duties; on a worldwide, perpetual, irrevocable, royalty-free basis to BMJ Publishing Group Ltd ("BMJ") its licensees and where the relevant Journal is co-owned by BMJ to the co-owners of the Journal, to publish the Work in this journal and any other BMJ products and to exploit all rights, as set out in our licence.

The Submitting Author accepts and understands that any supply made under these terms is made by BMJ to the Submitting Author unless you are acting as an employee on behalf of your employer or a postgraduate student of an affiliated institution which is paying any applicable article publishing charge ("APC") for Open Access articles. Where the Submitting Author wishes to make the Work available on an Open Access basis (and intends to pay the relevant APC), the terms of reuse of such Open Access shall be governed by a Creative Commons licence – details of these licences and which Creative Commons licence will apply to this Work are set out in our licence referred to above.

Other than as permitted in any relevant BMJ Author's Self Archiving Policies, I confirm this Work has not been accepted for publication elsewhere, is not being considered for publication elsewhere and does not duplicate material already published. I confirm all authors consent to publication of this Work and authorise the granting of this licence.

For peer review only

Merging Health and Police datasets for characterising interpersonal violence in the community of Khayelitsha, South Africa 2013-2015

Ardil Jabar[1], Tolu Oni[2,3], Leslie London[1], Annibale Cois[4,5], Richard Matzopoulos[1,5]

1. Division of Public Health Medicine, School of Public Health and Family Medicine, University of Cape Town, Cape Town, South Africa
2. Research Initiative for Cities Health and Equity (RICHE), School of Public Health and Family Medicine, University of Cape Town, Cape Town 7925 South Africa
3. MRC Epidemiology unit, University of Cambridge, Cambridge CB2 0QQ, UK
4. Division of Epidemiology and Biostatistics, School of Public Health and Family Medicine, University of Cape Town, Anzio Road, Cape Town 7925 South Africa
5. Burden of Disease Research Unit, South African Medical Research Council, Tygerberg 7505 Cape Town, South Africa

Corresponding author: Ardil Jabar, a.jabaroo@gmail.com

For peer review only

Abstract

Objectives

The Cardiff Model of data-sharing for violence prevention is premised on the idea that the majority of injury cases presenting at health facilities as a result of interpersonal violence will not be reported to the police. The aim of this study was to determine the concordance between violent crimes reported to the police with violence-related injuries presenting at health facilities in Cape Town, South Africa.

Methods

We conducted a retrospective analysis of secondary cross-sectional health and police data, from three health facilities and three police stations in the community of Khayelitsha, Cape Town. 781 cases of injuries arising from interpersonal violence seen at health facilities were matched with 739 violence-related crimes reported at police stations over five separate week-long sampling periods from 2013 to 2015. Personal identifiers, name and surname, were used to match cases.

Results

Of the 708 cases presenting at health facilities, 104 (14.7%) were matched with police records. The addition of non-reported cases of violence-related injuries from the health dataset to the police reported crime statistics, resulted in an 81.7% increase in potential total violent crimes over the reporting period. Compared to incidents reported to the police, those not reported were more likely to involve male patients (difference: +47.0%; p<0.001), and sharp object injuries (difference: +24.7%; p<0.001). Push/kick/punch injuries were more frequent among reporting than non-reporting patients (difference: +17.5%; p<0.001).

Conclusion

These findings suggest that the majority of injuries arising from interpersonal violence presenting at health facilities in Khayelitsha are not reported to the police. A data sharing model between health services and the police should be implemented to inform violence surveillance and reduction.

For peer review only - http://bmjopen.bmj.com/site/about/guidelines.xhtml

Strengths and limitations of this study

- To the best of our knowledge, this is the first study to attempt direct name matching between the health and the police to quantify the level of underreporting of interpersonal violence-related incidents
- This is the first attempt to validate the Cardiff model on the African continent
- One of the limitations of this study is that patients who presented to a health facility but only lodged criminal cases later outside of the one-week study periods over the three years would not be included in the police dataset and therefore not be included in the matching process.

For peer review only

For peer review only - http://bmjopen.bmj.com/site/about/guidelines.xhtml

Introduction

The Cardiff Model employs the strategic use of information from the health sector to improve policing and is an example of cross-sectoral collaboration. Developed by the Violence Research Group at Cardiff University, the model provides a way for communities to gain a clearer picture about where violence occurs by mapping both police and hospital violence data in combination (1). Application of the model in Cardiff in 2011 found that one half to two thirds of violence which results in hospital treatment is not known to the police (2). Subsequent research found that police recording of violence was limited to people reporting violent offenses, but that many of the injured who presented at emergency departments for treatment do not report the incident to police because they are either afraid of reprisals, do not want their own conduct scrutinised, or because they do not think the reporting will result in effective police action (3).

These findings have been replicated in other settings including England and the United States of America (USA), and the model is being considered for implementation in Australia and Jamaica (4)(5). Similarly, findings from the United States (US) Department of Justice National Crime Victimization Survey, a national household survey that sampled 701 000 individuals 12 years and older from 2006 to 2010, revealed that more than half (52%) of all violence incidents in the USA were not reported to law enforcement (6). We were unable to identify any similar studies in South Africa that compared the reporting of violence-related injuries to the police versus health facilities. However, we do know that violence is not always reported to the police. For example, in a community survey in Khayelitsha only 49% of violent incidents between family members were reported to the police (7).

The Western Cape Safety Plan, a policy document developed by the Western Cape Government, advocates the use of data and technology to understand violent crime patterns to inform the deployment of law enforcement resources and investigators accordingly and furthermore acknowledges research and analysis as an important component of its evidence-based policing (EBP) strategy (8). The policy document lends support to the implementation of EBP interventions such as the Cardiff Model locally.

For peer review only - http://bmjopen.bmj.com/site/about/guidelines.xhtml

The aim of this study was to determine the concordance between violent crimes reported to police stations and cases of injuries arising from inter-personal violence presenting to health facilities. Secondary objectives included the estimation of an adjusted crime profile (where police reported crime statistics included unreported violence from the Health data) and a comparison between patient populations that reported violence to the police with those that did not.

Methods

Study setting

Khayelitsha is a peri-urban low-income community of approximately 400 000 people located 30km from the Cape Town city centre (9). The homicide rate is well above the national average of 31 murders per 100 000 population at between 76 and 108 per 100 000 at Khayelitsha's different police stations (10). The community has mixed housing (informal and approximately 45% formal dwellings) and high unemployment (36%) (9) and is serviced by three police stations: Khayelitsha, Harare and Lingelethu West. Trauma cases are treated at three public-sector health facilities: Khayelitsha District Hospital (KDH), Site B and Michael Mapongwana Community Health Centre (Figure 1).

Study design

This was a retrospective cross-sectional analysis of secondary health and crime data from Khayelitsha for five 1-week-long periods collected between September 2013 and October 2015.

We assessed whether people presenting with injuries arising from interpersonal violence at health facilities went on to report these incidents to the police and whether victims reporting a crime to the police sought treatment at a health facility. We quantified this using personal identifier matching.

Data sources

The health data used in this study were originally collected by a non-profit research organisation, the Health Systems Trust (HST), for a study commissioned by the Western Cape Department of Health (DOH). Data were collected for five 1-week-long cross-section periods between 2013 to 2015. The health data were collected via face-to-face interviews with patients with violence-related injuries using a standardised data collection tool loaded on an electronic tablet, in the three health facilities (Figure 1) from 2013 to 2015 (11).

For peer review only - http://bmjopen.bmj.com/site/about/guidelines.xhtml

All emergency cases presenting to the health facilities were assessed for acute injuries which were classified according to cause of injury, viz: violence; transport; other unintentional injury or self-harm. Retrospective folder reviews of sexual assault cases that presented to the clinical forensic units at KDH during the corresponding study period were also included.

The police dataset included all violent crimes reported at all three police stations over the same time periods as the health dataset. This data was recorded in the national South African Police Services (SAPS) Crime Administration System (CAS) in Pretoria. A formal request to access the data was made to the SAPS National Research Division which was granted (Reference no. 3/34/2) on the 24th of November 2017.

Patient and Public Involvement

No patient involved

Variables

Cases presenting to health facilities for violence-related injuries included the following variables: hospital folder number as a personal identifier; age; gender; date and time of injury event; instrument used, type of violence; area (incident location); triage score; and alcohol use.

The inclusion of patient hospital numbers allowed for accessing patients' details on the Clinicom hospital system (a hospital patient tracking information system)(12), to identify patient names for matching with police data.

All acute injuries classified as person-on-person intentional violence were described according to the *external cause of injury* as per the International Classification of Disease (ICD) version 10 (ref), i.e.: sharp object (ICD10 code X99), blunt object (Y00), firearm, push/kick/punch (Y04), human bite (Y08), explosion (X09), choking/strangulation (X91), fire burn (X97), other burn, poisoning (X85), or unknown; and *type of violence* defined in the study as rape/sexual, gang-related, crime-related, interpersonal or unknown. "Gang-related' and "unknown" were collapsed into a combined 'interpersonal violence' category for purposes of comparison with the police data, which did not include these categories.

Cases recorded in the 'unknown' category included cases that recorded a specific cause of injury, where the type of violence was marked unknown and the victim-perpetrator category was also marked unknown. Patients treated for burns of unknown cause were included in the 'other burn' category.

For peer review only - http://bmjopen.bmj.com/site/about/guidelines.xhtml

The location of each assault was self-reported by patients attending health facilities according to ten predefined study areas (see Figure 1). The collection of incident location data allowed for geolocating each case in one of the ten study areas: Enkanini, Harare, Ilitha Park, Khaya, Kuyasa, Makaya, Makhaza, Site B, Site C, and Town 2 (Figure 1). Cases that were geolocated beyond the ten areas were excluded from the analysis.

The health data also included additional variables of interest that were not available in the police data. The triage colour provided a proxy for injury severity: viz: red (refer to major area for emergency management [immediate]); orange (refer to major area for urgent management [target time < 10 minutes]); yellow (refer to major area for urgent management [target time < 1 hour]); green [refer to designated area for non-urgent management (target time < 4 hours]) and blue (refer to doctor for certification [target time < 2 hours]). Alcohol and other drug use for each case was assessed by clinical judgement. Volume of consumption and consumption beyond six hours of the injury event was not assessed.

The police data included: name and surname as personal identifiers; age; gender; date and time of crime event; type of crime; instrument use; and incident location. Types of crimes included assault with the purpose to inflict grievous bodily harm; attempted murder; common assault; domestic violence; rape; sexual assault; and all types of robbery, which involve an interpersonal interaction with an implicit threat (i.e. common robbery; house robbery; robbery with a weapon or instrument other than a firearm; robbery and attempted robbery with a fire-arm; robbery and attempted robbery at business premises; carjacking [stealing an occupied motor-vehicle]) (13).

For purposes of comparison with the *type of violence* recorded in the health data, crime type was coded as follows: rape and sexual assault cases (rape/sexual); common assault , assault with the purpose to inflict grievous bodily harm, domestic violence attempted murder (interpersonal violence); all types of robbery, which involve an interpersonal interaction with an implicit threat (i.e. common robbery; house robbery; robbery with a weapon or instrument other than a firearm; robbery and attempted robbery with a fire-arm; robbery and attempted robbery at business premises; carjacking [stealing an occupied motor-vehicle] (crime-related).

The police recorded instrument use as a separate field and the categories were broadly consistent with the health data, as follows: sharp instrument, glass, key, knife, panga (bladed weapon), bottle, bottle head, screwdriver, (sharp object); blunt instrument, hammer, iron pipe, brick, stick, stone/brick (blunt object), firearm, pistol, revolver (firearm), head, fist, body part, feet, hands (push/kick/punch), belt, open hands (choking/strangulation); tyre, matches, petrol bomb (fire burn); electricity, ammunition (other burn); tik [methamphetamine], mandrax pills [quaalude], dagga [cannabis] (poisoning); and cell phone, motor vehicle, vehicle, cash, money, bank card, unknown (unknown).

For peer review only - http://bmjopen.bmj.com/site/about/guidelines.xhtml

Police incident location data were mapped based on self-reported data provided by victims of violence and were either geolocated using XY co-ordinates or recorded as a street address.

Case selection

Police records with multiple victims were expanded by replicating the common information (place, type of crime, etc.) to generate a single record for each victim in the final dataset.

Cases that were excluded from the health data included medical cases not related to injuries and injuries not caused by violence perpetrated on the victim by another subject (Condition 1); events that occurred outside the ten areas of Khayelitsha (Condition 2); cases in which the victim was younger than 16 years (Condition 3) (age of sexual consent in South Africa); and events that occurred outside the time period specified (Condition 4).

Cases that were removed from the police data include cases other than the types of crimes described above (Condition 1); events that occurred outside the ten areas of Khayelitsha (Condition 2); cases in which the victims were younger than 16 years (Condition 3) and events that occurred outside the time period specified (Condition 4).

Data analysis

The two datasets were captured in Microsoft Excel, and imported into the R Statistical environment v. 3.5.2 for analysis (14). Two-side statistical tests with a conventional 5% cut-off for statistical significance were conducted to assess differences in characteristics between injury patients from the health database and crime victims from the police database, and between reporting and non-reporting patients in the health database.

χ^2 tests were used to evaluate differences in the distribution of categorical variables. When comparing the police dataset with the health dataset, a robust estimator of the test variance was used to take into account the partial overlapping of the two samples (15).

Cases from health facilities were matched with police data using name, surname, gender and age as matching keys. Some latitude was accorded to allow for spelling variations and approximate recording of ages. This was done as follows: patient hospital numbers were entered into the Groote Schuur Hospital (GSH) Clinicom database to retrieve the patient name. The Levenshtein distance (a string metric for measuring differences between two sequences of characters (16)) was calculated for each patient between his/her name and each of the names present in the police dataset, and the police record(s) was selected corresponding to the minimum distance as "candidate" matches. In the second step (exclusion of unlikely matches) the potential matches

For peer review only - http://bmjopen.bmj.com/site/about/guidelines.xhtml

generated from the first step were retained for subsequent analysis if (i) names were identical between the two datasets; or (ii) the Levenshtein distance was 1, gender was the same and reported ages differed less than 5 years; or (iii) the Levenshtein distance was > 1, gender was the same and reported ages differed less than 3 years. In the third step (manual selection) the potential matches resulting from the previous step were manually inspected to verify the actual correspondence between victim names and the consistency of the reported time of the violent episode, which led to the elimination of further cases.

An estimation of an adjusted crime profile was done by adding unreported patient violence seen at health facilities to the police reported crime statistics. The patients who reported violence to police ("reporting patients") and the patients who did not report the violence to the police ("non-reporting patients") were inferred from the matching process, and are described with regards to demographic characteristics (age and gender), instrument use, type of offence, triage code and alcohol use.

Ethics

Ethical permission to conduct this research was granted by the University of Cape Town Human Research Ethics Committee (UCT HREC 861/2016) and the South African Police Services Research Division (3/34/2). With regard to the HST data, informed consent was obtained only from violence-related injury cases and where given, these individuals were interviewed by the data collectors, with only violence-related injury cases having the full data collection tool completed. Informed consent was granted by Groote Schuur Hospital for permission to access the Clinicom patient database in order to complete the personal identifier matching.

Results

Summary of included data

The health dataset initially included a total number of 9608 records of patients who presented at the three facilities during the five time periods considered. After applying exclusions (medical conditions, injury not due to interpersonal violence, injury occurring out the study area or study period and child injury), the final dataset included 781 records (Figure 2).

The police dataset initially included a total number of 2161 records of criminal episodes. After applying exclusions (cases occurring outside the five study periods, cases not included in the types of included crimes list, cases where the victim was younger than 16, cases where the

For peer review only - http://bmjopen.bmj.com/site/about/guidelines.xhtml

incident location was outside one of the ten study areas) the final dataset included 739 records (Figure 2). 358 police records had missing values on gender and age.

Patients seen at health facilities (69.8%) and victims of violence (51.4%) were both more frequently male (Table 1). The mean age for patients was 29.9 for patients and 35.2 for victims of violence. The most common age ranges for both datasets were 25-34 (health 42.2%, police 34.1%), 16-24 (health 33.7%, police 22.3%) and 35-44 (health 15.1%, police 18.9%). At health facilities violence peaked on Sundays (33.7%), whereas violent crimes were most frequently reported to the police on Saturdays (22.3%).

The most common instrument described in the police database was an assault with body part (push/kick/punch) followed by unknown, firearm, sharp object and blunt object. At health facilities, the most common instrument use was sharp objects, followed by an assault with body part (push/kick/punch), blunt objects, unknown and firearms. Sharp objects comprised 60.7% for health presentations versus 12% for police presentations while body part was 41.8% for police station presentation, while only 14.7% for health.

Site B recorded the most cases in both health (36.2%) and police (32.9%) datasets with Ilitha Park (2.7%) and Enkanini (1.2%) recording the fewest cases respectively.

Table 1. Distribution of cases recorded in health facilities and by the police according to gender, age, day of the week, time of day, instrument use, type of violence, incident location, triage score and alcohol/drug use

Variable	HEALTH data (N=781)	POLICE data (N=739)
	n (%)	n (%)
**Name Surname completeness**	**708 (90.7)**	**739 (100)**
**Gender***		
Males	**545 (69.8)**	**196 (51.4)**
Females	**236 (30.2)**	**185 (48.6)**
**Age (years)***		
16-24	263 (33.7)	85 (22.3)
25-34	330 (42.2)	130 (34.1)
35-44	118 (15.1)	72 (18.9)

For peer review only - http://bmjopen.bmj.com/site/about/guidelines.xhtml

45-54	46 (5.9)	56 (14.7)
55+	24 (3.1)	38 (10.0)
**Day of week**		
Monday	95 (12.2)	103 (13.9)
Tuesday	50 (6.4)	72 (9.7)
Wednesday	48 (6.1)	75 (10.1)
Thursday	45 (5.8)	64 (8.7)
Friday	77 (9.8)	100 (13.5)
Saturday	203 (26.0)	165 (22.3)
Sunday	263 (33.7)	160 (21.7)
**Time of day**		
00h00:01h59	65 (8.3)	60 (8.1)
02h00:03h59	42 (5.4)	29 (3.9)
04h00:05h59	40 (5.1)	42 (5.7)
06h00:07h59	65 (8.3)	71 (9.6)
08h00:09h59	52 (6.7)	34 (4.6)
10h00:11h59	64 (8.2)	49 (6.6)
12h00:13h59	73 (9.3)	45 (6.1)
14h00:15h59	63 (8.1)	68 (9.2)
16h00:17h59	76 (9.7)	67 (9.1)
18h00:19h59	57 (7.3)	69 (9.3)
20h00:21h59	101 (12.9)	119 (16.1)
22h00:23h59	83 (10.6)	86 (11.6)
**Instrument used**		
Sharp object	474 (60.7)	89 (12.0)
Blunt object	112 (14.3)	70 (9.5)
Firearm	29 (3.7)	89 (12.0)
Push/kick/punch	115 (14.7)	309 (41.8)
Human bite	6 (0.8)	2 (0.3)
Choking/strangulation	2 (0.3)	8 (1.1)
Fire burn	2 (0.3)	5 (0.7)
Other burn	3 (0.4)	2 (0.3)
Poisoning	0 (0.0)	56 (7.6)
Unknown	38 (4.9)	109 (14.7)
**Type of violence**		

For peer review only

Rape/sexual	16 (2.0)	67 (9.1)
Crime-related	290 (37.0)	250 (33.8)
Interpersonal	266 (44.8)	422 (57.1)
Gang-related	84 (10.7)	
Unknown	125 (16.0)	**
**Area**		
Enkanini	41 (5.2)	9 (1.2)
Harare	85 (10.9)	66 (8.9)
Ilitha Park	21 (2.7)	42 (5.7)
Khaya	30 (3.8)	74 (10.0)
Kuyasa	39 (5.0)	45 (6.1)
Makaya	43 (5.5)	37 (5.0)
Makhaza	85 (11.0)	96 (13.0)
Site B	283 (36.2)	243 (32.9)
Site C	118 (15.1)	91(12.3)
Town 2	36 (4.6)	36 (4.9)
**Triage scores** ***		
Red	65 (8.3%)	-
Orange	251 (32.1%)	-
Yellow	380 (48.6%)	-
Green	68 (8.7%)	-
Blue	1 (0.12)	-
Unknown	18 (2.2%)	-
**Alcohol use** ***		
Yes	444 (56.8%)	-
No	284 (36.4%)	-
Unknown	53 (6.8%)	-

* 358 police records had missing values on gender and age

** For type of violence, the police data did not record an ‘unknown or gang-related’ category

*** Triage score and alcohol use data were only available for the health dataset

For peer review only

For peer review only - http://bmjopen.bmj.com/site/about/guidelines.xhtml

Concordance

Among the 708 patients being treated for violence-related injuries at health facilities for which the name and surname could be retrieved from the Clinicom database, only 104 reported the incident to the police, which equates to a matching ratio of 14.7% (Figure 3).

Adjusted crime profile

Combining unreported cases from health records to the police reported crime statistics provides an adjusted crime profile (Table 2). The addition of patient violence from the health data to the police reported crime statistics, resulted in an 81.7% increase in potential total violent crimes over the reporting period. Males represent the majority in the combined (65.0%) dataset. In terms of day of week, Saturday and Sunday were the busiest days in the combined dataset with Thursday being the quietest.

Push/kick/punch (41.8% and 40.3%) is the most common instrument used in the police and combined dataset respectively, with the commonest type of violence being interpersonal violence for both (57.1 and 58.1%). Site B, Site C and Makhaza ranked amongst the top three for both datasets with Enkanini (1.5%) similarly recording the fewest cases.

Notable shifts in ranking when combining the datasets were found in day of week (Saturday ranked 1 in police versus 2 in combined dataset), time of day, instrument used (push/kick/punch ranked 1 in police data [41.8%], and sharp object ranked 1 in combined data [35.4%]), and area where the positions for Makhaza and Site C were changed in the top 3 ranked areas.

For peer review only - http://bmjopen.bmj.com/site/about/guidelines.xhtml

For peer review only

Table 2. Adjusted crime profile comparing police versus police and health combined data

Variable	POLICE data (N=739)		Combined Data (N=1343)	
	Rank	n (%)	Rank	n (%)
**Gender***				
Males	1	196 (51.4)	1	640 (65.0)
Females	2	185 (48.6)	2	345 (35.0)
**Age (years)***				
16-24	2	85 (22.3)	2	274 (27.8)
25-34	1	130 (34.1)	1	394 (40.0)
35-44	3	72 (18.9)	3	173 (17.6)
45-54	4	56 (14.7)	4	87 (8.8)
55+	5	38 (10.0)	5	57 (5.8)
**Day of week**				
Monday	3	103 (13.9)	3	178 (13.2)
Tuesday	6	72 (9.7)	6	107 (8.0)
Wednesday	5	75 (10.1)	5	109 (8.1)
Thursday	7	64 (8.7)	7	96 (7.1)
Friday	4	100 (13.5)	4	155 (11.5)
Saturday	1	165 (22.3)	2	320 (23.8)
Sunday	2	160 (21.7)	1	378 (28.1)
**Time of day**				
00h00:01h59	7	60 (8.1)	6	109 (8.1)
02h00:03h59	12	29 (3.9)	11	63 (4.7)
04h00:05h59	10	42 (5.7)	10	70 (5.2)
06h00:07h59	3	71 (9.6)	4	120 (8.9)
08h00:09h59	11	34 (4.6)	9	79 (5.9)
10h00:11h59	8	49 (6.6)	8	97 (7.2)
12h00:13h59	9	45 (6.1)	7	101 (7.5)
14h00:15h59	5	68 (9.2)	5	113 (8.4)
16h00:17h59	6	67 (9.1)	3	131 (9.7)

For peer review only - http://bmjopen.bmj.com/site/about/guidelines.xhtml

18h00:19h59	4	69 (9.3)	6	109 (8.1)
20h00:21h59	1	119 (16.1)	1	198 (14.7)
22h00:23h59	2	86 (11.6)	2	153 (11.4)
**Instrument used**				
Sharp object	3	89 (12.0)	1	476 (35.4)
Blunt object	4	70 (9.5)	5	155 (11.5)
Firearm	3	89 (12.0)	4	111 (8.3)
Push/kick/punch	1	309 (41.8)	2	383 (28.5)
Human bite	8	2 (0.3)	9	5 (0.4)
Choking/strangulation	6	8 (1.1)	7	9 (0.7)
Fire burn	7	5 (0.7)	8	7 (0.5)
Other burn	8	2 (0.3)	10	5 (0.4)
Poisoning	5	56 (7.6)	6	56 (4.2)
Unknown	2	109 (14.7)	3	136 (10.1)
**Type of violence**				
Rape/sexual	3	67 (9.1)	3	74 (5.5)
Crime-related	2	250 (33.8)	2	489 (36.4)
Interpersonal **	1	422 (57.1)	1	780 (58.1)
**Area**				
Enkanini	10	9 (1.2)	8	41 (3.1)
Harare	5	66 (8.9)	4	129 (9.6)
Ilitha Park	7	42 (5.7)	7	59 (4.4)
Khaya	4	74 (10.0)	4	96 (7.1)
Kuyasa	6	45 (6.1)	5	74 (5.5)
Makaya	8	37 (5.0)	6	70 (5.2)
Makhaza	2	96 (13.0)	3	163 (12.1)
Site B	1	243 (32.9)	1	461 (34.3)
Site C	3	91(12.3)	2	188 (14.0)
Town 2	9	36 (4.9)	7	62 (4.6)

*358 police records had missing values on gender and age

** For purposes of comparison, 'gang-related' cases from the health data were included as 'interpersonal violence' cases. Additionally, the 'unknown 'category was included as 'interpersonal violence' for purposes of comparison and ranking.

For peer review only - http://bmjopen.bmj.com/site/about/guidelines.xhtml

Predictors of non-reporting

Male patients (73.5%) that were stabbed (64.1%), had alcohol use confirmed or suspected (58.9%) and experienced crime (39.6%) or gang-related incidents (11.4%) were less likely to report their injuries to the police. Patients suffering sharp object (39.4%) or push/kick/punch injuries (29.8%) or sexual assault/rape (8.7%) were more likely to report their injuries (Table 3).

No statistically significant differences were present regarding age and severity of the injury (as measured by the triage code). There were minimal differences in location and timing of the violent episode between reporting and non-reporting patients. The one significant difference in timing of the violence episode was the almost double number of episodes reported on Sunday among non-reporting patients when compared to reporting patients (36.1% vs. 20.2%, p=0.017).

For peer review only

Table 3. Difference in demographic and clinical characteristics and in type of offence between patients who did/did not report the violence to police

**Variable**	Patients who **did not report** the violence: N = 604	Patients who **reported** the violence: N = 104	**All** patients: * N = 708	
	**n (%)**	**n (%)**	**n (%)**	**p-value****
**Gender**				
Male	444 (73.5%)	44 (42.3%)	488 (68.9%)	
Female	160 (26.5%)	60 (57.7%)	220 (31.1%)	**<0.001**
**Age (years)**				
16-24	188 (31.2)	41 (39.4)	229 (32.4)	
25-34	264 (43.8)	34 (32.7)	298 (42.1)	
35-44	101 (16.7)	16 (15.4)	117 (16.5)	**0.017**
45-54	31 (5.1)	12 (11.5)	43 (6.1)	
55+	20 (3.2)	1 (1.0)	21 (2.8)	
**Day of week**				
Monday	75 (12.4)	14 (13.5)	89 (12.6)	**0.017**
Tuesday	35 (5.8)	12 (11.5)	47 (6.6)	

For peer review only - http://bmjopen.bmj.com/site/about/guidelines.xhtml

Wednesday	34 (5.6)	10 (9.6)	44 (6.2)	
Thursday	32 (5.3)	5 (4.8)	37 (5.2)	
Friday	55 (9.1)	15 (14.4)	70 (9.9)	
Saturday	155 (25.7)	27 (26)	182 (25.7)	
Sunday	218 (36.1)	21 (20.2)	239 (33.8)	
**Time of day**				
00h00:01h59	49 (8.1)	8 (7.7)	57 (8.1)	0.267
02h00:03h59	34 (5.6)	6 (5.8)	40 (5.6)	
04h00:05h59	28 (4.6)	6 (5.8)	34 (4.8)	
06h00:07h59	49 (8.1)	10 (9.6)	59 (8.3)	
08h00:09h59	45 (7.5)	5 (4.8)	50 (7.1)	
10h00:11h59	48 (7.9)	7 (6.7)	55 (7.8)	
12h00:13h59	56 (9.3)	12 (11.5)	68 (9.6)	
14h00:15h59	45 (7.5)	15 (14.4)	60 (8.5)	
16h00:17h59	64 (10.6)	4 (3.8)	68 (9.6)	
18h00:19h59	40 (6.6)	8 (7.7)	48 (6.8)	
20h00:21h59	79 (13.1)	12 (11.5)	91 (12.9)	
22h00:23h59	67 (11.1)	11 (10.6)	78 (11)	
**Instrument**				
Sharp object	387 (64.1)	41 (39.4)	428 (60.5)	**<0.001**
Blunt object	85 (14.1)	18 (17.3)	103 (14.5)	
Firearm	22 (3.6)	5 (4.8)	27 (3.8)	
Push/kick/punch	74 (12.3)	31 (29.8)	105 (14.8)	
Human bite	3 (0.5)	1 (1)	4 (0.6)	
Choking/strangulation	1 (0.2)	1 (1)	2 (0.3)	
Fire burn	2 (0.3)	0 (0)	2 (0.3)	
Other burn	3 (0.5)	0 (0)	3 (0.4)	
Poisoning	0 (0)	0 (0)	0 (0)	
Unknown	27 (4.5)	7 (6.7)	34 (4.8)	
**Type of violence**				
Rape/sexual	7 (1.2)	9 (8.7)	16 (2.3)	**<0.001**
Crime-related	239 (39.6)	26 (25.0)	265 (37.4)	
Interpersonal	199 (33.0)	45 (43.2)	244 (34.5)	
Gang-related	69 (11.4)	6 (5.8)	75 (10.6)	
Unknown	90 (14.9)	18 (17.3)	108 (15.3)	
**Area**				

For peer review only - http://bmjopen.bmj.com/site/about/guidelines.xhtml

Enkanini	32 (5.3)	4 (3.8)	36 (5.1)	0.689
Harare	63 (10.4)	14 (13.5)	77 (10.9)	
Ilitha Park	17 (2.8)	1 (1)	18 (2.5)	
Khaya	22 (3.6)	4 (3.8)	26 (3.7)	
Kuyasa	29 (4.8)	6 (5.8)	35 (4.9)	
Makaya	33 (5.5)	7 (6.7)	40 (5.6)	
Makhaza	67 (11.1)	13 (12.5)	80 (11.3)	
Site B	218 (36.1)	38 (36.5)	256 (36.2)	
Site C	97 (16.1)	10 (9.6)	107 (15.1)	
Town 2	26 (4.3)	7 (6.7)	33 (4.7)	
**Triage code**				
Red	47 (7.8)	7 (6.7)	54 (7.6)	0.278
Orange	204 (33.8)	23 (22.1)	227 (32.1)	
Yellow	290 (48)	58 (55.8)	348 (49.2)	
Green	53 (8.8)	8 (7.7)	61 (8.6)	
Blue	1 (0.2)	0 (0)	1 (0.1)	
Unknown	9 (1.5)	8 (7.7)	17 (2.4)	
**Alcohol use**				
Yes/Suspected	356 (58.9)	49 (47.1)	405 (57.2)	**0.012**
No	205 (33.9)	50 (48.1)	255 (36)	
Unknown	43 (7.1)	5 (4.8)	48 (6.8)	

* For which the name and surname could be retrieved from the Clinicom database;

** Statistical test for the existence of differences between reporting and not-reporting patients.

Discussion

One of the key findings of a 2014 systematic review of the feasibility and effectiveness of community-level interventions involving emergency department data sharing to reduce alcohol-related violence, was that data sharing protocols can be cheaply and easily implemented into modern Emergency Department (ED) triage systems (17). There was general consensus among studies that this could be done with minimal cost, staff workload burden and impact to patient safety. Additionally, there was minimum risk to patient service and anonymity, risk of harm displacement to other licensed venues or increase to length of patient stay (17). The Cardiff Model is cost-effective with one study finding that for every $1 spent, nearly $15 in health system and over $19 in criminal justice system costs are saved (18).

For peer review only - http://bmjopen.bmj.com/site/about/guidelines.xhtml

To the best of our knowledge, this is the first study to attempt direct name matching between the health and the police to quantify the level of underreporting of interpersonal violence-related incidents. The matching method using personal identifiers suggest that only a small fraction (approximately 15 %) of patients being treated for interpersonal violence at local health facilities will go on to report these as crimes to local police stations within a week. Conversely, only a small proportion of those who report a violent crime to the police go on to present to a health facility with an injury within a week.

The proportion of cases in this study of injuries arising from interpersonal violence which are not reported to the police (approximately 82%), is consistent with the findings of the Cardiff model which found that one half to two thirds of violence which results in hospital treatment is not known to the police (2). This finding is much lower when compared with other regions including in developed countries. According to a 2016 U.S. Department of Justice report, many crimes go unreported to law enforcement, including 53% of violent crime in 2015; 58% of simple assaults in 2015 and 43% of violent crime involving an injury in 2015 (19).

Possible reasons for the high level of underreporting (30%+ discrepancy between the level of under reporting in this study [82%) and the Cardiff model [50%]), can be found in the report of the 2012 Khayelitsha Commission of inquiry (also known as the O'Regan/Pikoli Commission) which investigated allegations of police inefficiency in Khayelitsha and the breakdown in relations between the Khayelitsha community and the police. Poor policing conditions characterised by police in the area being generally lower-ranked, poorly equipped, few in number, underqualified and reluctant to open cases, led to breakdown in trust with the community resulting in the lack of reporting (20). Other possible reasons for underreporting include the fear of reprisal from perpetrators, being turned away by police when presenting at the police station to report a crime, stigma/shame, or not perceiving an injury arising from interpersonal violence as a crime.

Type of injury

The majority of police reported crimes are related to assault with body part (push/kick/punch). Victims of knife crimes represented in the health data, which often cause worse injuries requiring medical intervention, are not reporting these incidents to police. Gang/crime-related episodes were more common among non-reporting patients, which may be due to a lack of confidence in the police's ability to protect them from reprisal, or if the victim perceived the gangs as being protected by or working with the police, in a known context of mistrust of police in the community of Khayelitsha (20). Additionally, the underreporting of knife wielding gang-related violence incidents in police statistics may be a contributing factor. This is supported by the lower proportion of younger male victims (age 16-24) reporting crime compared to those receiving treatment for an injury arising from interpersonal violence (31.2% vs 39.4%, P=0.017).

For peer review only - http://bmjopen.bmj.com/site/about/guidelines.xhtml

The scale in difference of sharp object injuries presenting at police (89 cases) and health facilities (474), means that 385 victims of violence treated for a sharp object injury at a health facility did not go on to report the crime to the police, which if added to the official police statistics would represent a 52% increase in all violent crimes reported. This provides evidence for an excess burden of injuries arising from interpersonal violence which is not being reported to police – a situation that is consistent with the premise of the Cardiff model.

Limitations

One of the limitations of this study is that approximately 10% of health records lacked names and were thus excluded from the matching process. A 2013 South African study in missing medical records found that up to 8% of hospital folders had the wrong names and that 17% of hospital folders had names without folder numbers (21).

No cases of poisoning were recorded in the health data. The current referral practice is for Michael M and Site B to refer their poisoning cases to KDH. At KDH most poisoning cases are triaged to the Medical department and thus these cases would not be recorded in the health data. The exception being cases of poisoning due to a corrosive substance such as bleach, which would be triaged to the Trauma/Surgical department due to the possible need for a surgical therapeutic intervention. It is a recommendation of study authors that the poisoning data from medical emergencies be included to inform this work going forward.

One of the weaknesses of this study is that patients who presented to a health facility but only lodged criminal cases later outside of the one-week study periods over the five years would not be included in the police dataset and therefore not be included in the matching process. Similarly, if victims of injuries arising from interpersonal violence presented to the police first and did not present to the health facilities for treatment within the one-week study period, they were also not included in the matching process. The inclusion of these late reporting cases could decrease the overall proportion of interpersonal violence-related injuries that were reported to the police. Finally, patients experiencing inter-personal violence and who sought private health care or presented directly to the hospitals outside of the district (such as the tertiary referral hospital, Tygerberg Hospital) would not be included in the analyses. This may increase or decrease the estimate of concordance depending on whether these patients reported to the police.

Implications for policy and interventions

For peer review only - http://bmjopen.bmj.com/site/about/guidelines.xhtml

Murder and violence are often linked to alcohol with a 2016 analysis of murder dockets finding that in up to 48% of murders, the victim or perpetrator was intoxicated (22). Alcohol use was higher in patients who did not report violence than patients who did report violence to the police. This is of public health importance as alcohol has been identified as a major risk factor for and predictor of violence and is amenable to policy change to reduce alcohol harms. The current evidence base for measuring alcohol use within crime data will underestimate the extent of the problem because of the underreporting in routine crime statistics highlighted by this research.

Furthermore, with regard to local municipal alcohol policy on liquor outlet licences (23), reasons for limitation of liquor outlet licences may include, 'safety and social problems and/or concerns arising from alcohol abuse, alcohol related crimes, illegal criminal activity, etc, using data derived from the South African Police Service'. The local alcohol policy is heavily reliant on the SAPS data to guide license approvals, which this research has shown to be incomplete and flawed in its underestimation of interpersonal violence at the community level. Alcohol policy decisions made by Government at any level (local, district, provincial and national) that is informed by this data, may aggravate alcohol related harms.

The level of underreporting exposed by the merging of health and police datasets and matching exercise may speak to a larger question of police resourcing within Khayelitsha. The Khayelitsha commission of inquiry highlighted the insufficient police human resources within police stations in Khayelitsha as one of the reasons for community mistrust of the police(20). The current SAPS resource allocation model is based on the Theoretical Human Resource Requirement (THRR) and fixed establishment (FE) approach which was developed in 2012 (24) . The tool takes into consideration annual reported crime per police station (24) which suggests that current police human resources deployed in Khayelitsha may be severely under resourced to deal with the 'real' burden of violence, when unreported health data is considered.

The validity of self-reported drinking using ED data from 16 countries reported findings suggesting that the overall validity of self-reported alcohol consumption before injury is high (88%) compared with a blood alcohol concentration (BAC) estimate obtained at the time of admission to the ED, with a caveat that demographic, drinking and injury characteristics may affect validity(25).

This study provides evidence showing that interpersonal violence at the community level cannot be estimated accurately relying solely on reported crimes to the police services. Injuries arising from interpersonal violence treated at health facilities must be included to more accurately define the level of interpersonal violence within a community.

For peer review only - http://bmjopen.bmj.com/site/about/guidelines.xhtml

Implementation of the Hospital Emergency Centre Tracking Information System (HECTIS), an electronic data collection system for Emergency Departments (ED), is currently underway at the Michael Mapongwana CHC and KDH. This will improve the monitoring of injuries arising from interpersonal violence (the majority of which are not reported to the police) and provide a method for the evaluation of violence prevention interventions. Additionally, this will provide the data infrastructure required for the potential implementation of the Cardiff model locally.

Conclusion

The matching exercise described above represents the first attempt within South Africa to match patients at health facilities treated for injuries arising from interpersonal violence with those of victims reporting violent crimes to police stations within the same community and time period. This matching study suggests that the majority of injuries arising from interpersonal violence presenting at health facilities in Khayelitsha are not reported to the police in Khayelitsha.

This study has broader implications regionally and nationally for the surveillance of injuries arising from interpersonal violence, for the police definition and surveillance of community interpersonal violence, for community policing intelligence development (improving the configuration of violence heat maps on a real time basis) and finally for police resource utilisation and distribution, which should, in turn, impact positively on reducing crime and violence in the community, and reduce the burden on the Health services. The implementation of the Cardiff data sharing model should be adopted to inform violence reduction locally.

Ethics

Ethical permission to conduct this research was granted by the University of Cape Town Human Research Ethics Committee (UCT HREC 861/2016) and the South African Police Services Research Division (Application no. 3/34/2). With regard to the HST data, informed consent was obtained only from acute injury cases and where given, these individuals were interviewed by the data collectors, with only acute injury cases having the full data collection tool completed. Informed consent was granted by Groote Schuur Hospital for permission to access the Clinicom patient database.

Funding

For peer review only

This work was supported by the International Development Research Centre (IDRC), Canada, and the UK Government's Department for International Development (DFID) (Grant number 107329–001) and the Western Cape Government Department of Health (Grant number WCDOH428/2013). The degree from which this study emanated was funded by the South African Medical Research Council (SAMRC) under the SAMRC Clinician Researcher (MD PhD) Scholarship Programme. RM is funded by the SAMRC. TO is supported by the National Institute for Health Research (NIHR) (16/137/34) using UK aid from the UK Government to support global health research. The views expressed in this publication are those of the authors and not necessarily those of the NIHR or the UK Department of Health and Social Care or SAMRC.

Figure 1. Participating health facilities and police stations in relation to study areas in the community of Khayelitsha

Figure 2. Case selection flow chart for health and police datasets

Figure 3. Personal identifier matching between datasets

For peer review only

References

1. CDC. Community Guidance for Violence Prevention Cardiff Model Toolkit [Internet]. Atlanta; 2018. Available from: https://www.cdc.gov/violenceprevention/fundedprograms/cardiffmodel/toolkit.html

2. Florence C, Shepherd J, Brennan I, Simon T. Effectiveness of anonymised information sharing and use in health service, police, and local government partnership for preventing violence related injury: experimental study and time series analysis. BMJ. 2011;342:d3313.

3. Group violence research. Cardiff Model for Violence Prevention Implementation of the Model in the UK International [Internet]. Vol. 44. Cardiff; 2018. Available from: https://static1.squarespace.com/static/57875c16197aea2902e3820e/t/5a03139f71c10ba51be806ee/1510151075166/vrg+cardiff+model+briefing+web.pdf

4. Kollar L, Sumner S, Bartholow B, Wu D, Moore J, Mays E, et al. Building capacity for injury prevention : a process evaluation of a replication of the Cardiff Violence Prevention Programme in the Southeastern USA. Inj Prev. 2019;1–8.

5. Boyle A a, Snelling K, White L, Ariel B, Ashelford L. External validation of the Cardiff model of information sharing to reduce community violence: natural experiment. Emerg Med J [Internet]. 2013;30(12):1020–3. Available from: http://www.ncbi.nlm.nih.gov/pubmed/23184923

6. Langton L, Berzofsky M, Krebs C, Smily-McDonald H. Victimizations not reported to police [Internet]. Bureau of Justice statistics. [cited 2017 Feb 17]. Available from: http://www.bjs.gov/content/pub/pdf/vnrp0610.pdf.2012

7. Cassidy T, Lloyd S, Bowman B, Myers J, Parry C, Makanga T, et al. Authors : [Internet]. Cape Town; 2013. Available from: https://open.uct.ac.za/bitstream/handle/11427/28271/Cassidy_Survey violence alcohol.pdf?sequence=3

8. Government WC. Western Cape Safety Plan Working Document. Cape Town; 2019.

For peer review only - http://bmjopen.bmj.com/site/about/guidelines.xhtml

9. City of Cape Town. City of Cape Town – 2011 Census Suburb Khayelitsha [Internet]. Cape Town; 2011. Available from: https://www.capetown.gov.za/en/stats/2011CensusSuburbs/2011_Census_CT_Suburb_Khayelitsha_Profile.pdf

10. Barolsky V. Violence in Khayelitsha: finding a way out. Cape Town; 2014.

11. Mureithi L, Africa A, Wandai M, Schaik N Van, Naledi T, Matzopoulos R, et al. Injury Morbidity Surveillance in Nyanga and Khayelitsha in the Western Cape. Vol. 27. Cape Town; 2016.

12. Government WC. New Patient Administrative (AR) and Billing system at Prince Albert Hospital [Internet]. Western Cape Government news. 2020 [cited 2020 Nov 17]. Available from: https://www.westerncape.gov.za/news/new-patient-administrative-ar-and-billing-system-prince-albert-hospital

13. SAPS. SAPS crime defintions. SAPS Website. 2019.

14. R Core Team. R: A language and environment for statistical computing [Internet]. R Foundation for Statistical Computing. 2013 [cited 2020 Sep 3]. Available from: http://www.r-project.org/

15. Zeileis A. Object-Oriented Computation of Sandwich Estimators. J Stat Softw. 2006;16(9):1–16.

16. Doan A, Halevy A, Ives Z. Principles of Data Integration. 1st ed. Morgan Kaufmann; 2012. 95–119 p.

17. Droste N, Miller P, Baker T. Review article: Emergency department data sharing to reduce alcohol-related violence: A systematic review of the feasibility and effectiveness of community-level interventions. Emerg Med Australas [Internet]. 2014;26(4):326–35. Available from: http://doi.wiley.com/10.1111/1742-6723.12247

18. Holder, Yvette et al. Injury Surveillance Guidelines. Geneva; 2001.

19. Morgan R, Kena G. Criminal Victimization,2016:Revised [Internet]. Bureau of Justice Statistics. 2018. Available from: https://www.bjs.gov/content/pub/pdf/cv16.pdf

20. WCG. Report of the Commission of Inquiry into Allegations of Police Inefficiency and a Breakdown in Relations between SAPS and the Community of Khayelitsha. Cape Town; 2014.

21. Wegner L, Rhoda A. Missing medical records : an obstacle to archival survey-research in a rural community in South Africa. South African J Physiother. 2013;15–9.

22. SAPS. Crime statistics South Africa [Internet]. Cape Town; 2016. Available from: https://www.saps.gov.za/services/crimestats.php

For peer review only - http://bmjopen.bmj.com/site/about/guidelines.xhtml

23. Municipality D. Drakenstein Municipality Liquor Outlet and Tavern draft Policy [Internet]. Cape Town: Drakenstein Municipality; 2020. Available from: http://www.drakenstein.gov.za/docs/Documents/DM Liquor Policy - 3rd draft_20200723.pdf

24. Government WC. Analyses of Human Resource Allocation for South African Police Service with specific reference to the Western Cape. Cape Town; 2017.

25. Cherpitel C, Ye Y, Bond J, G B, Macdonald S, Stockwell T, et al. Validity of self-reported drinking before injury compared with a physiological measure: cross national analysis of Emergency department data from 16 countries. 2006.

For peer review only

For peer review only - http://bmjopen.bmj.com/site/about/guidelines.xhtml

Figure 1

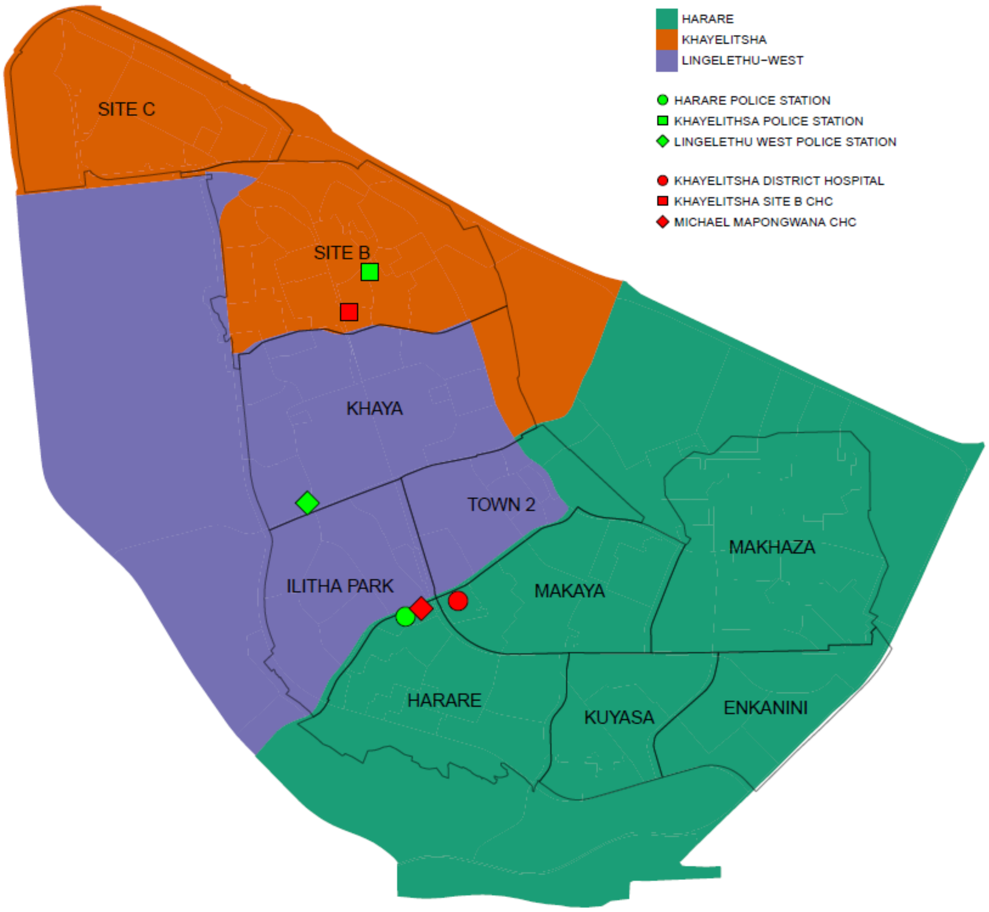

Figure 1. Participating health facilities and police stations in relation to study areas in the community of Khayelitsha

Figure 2

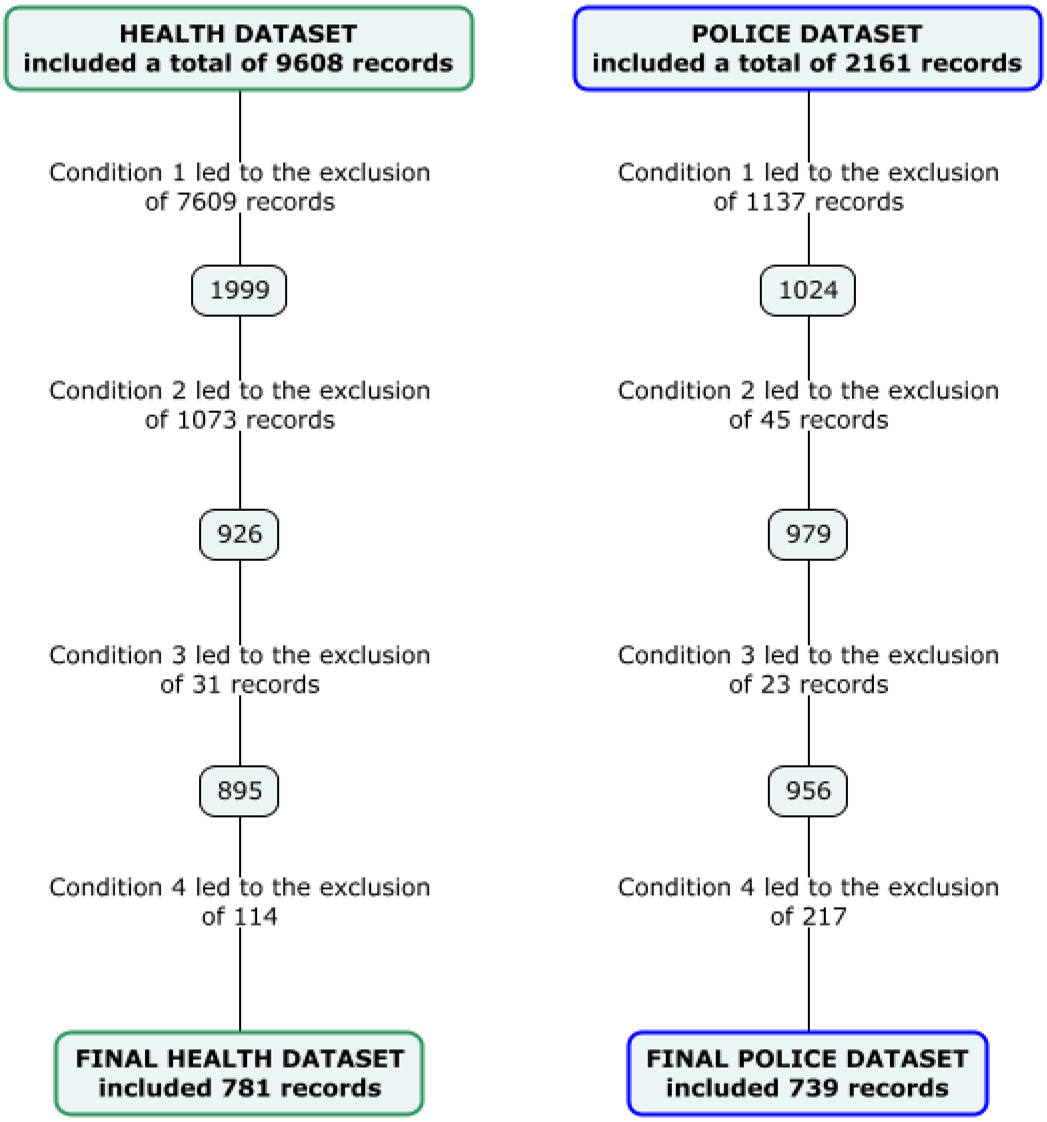

Figure 2. Case selection flow chart for health and police datasets

Figure 3

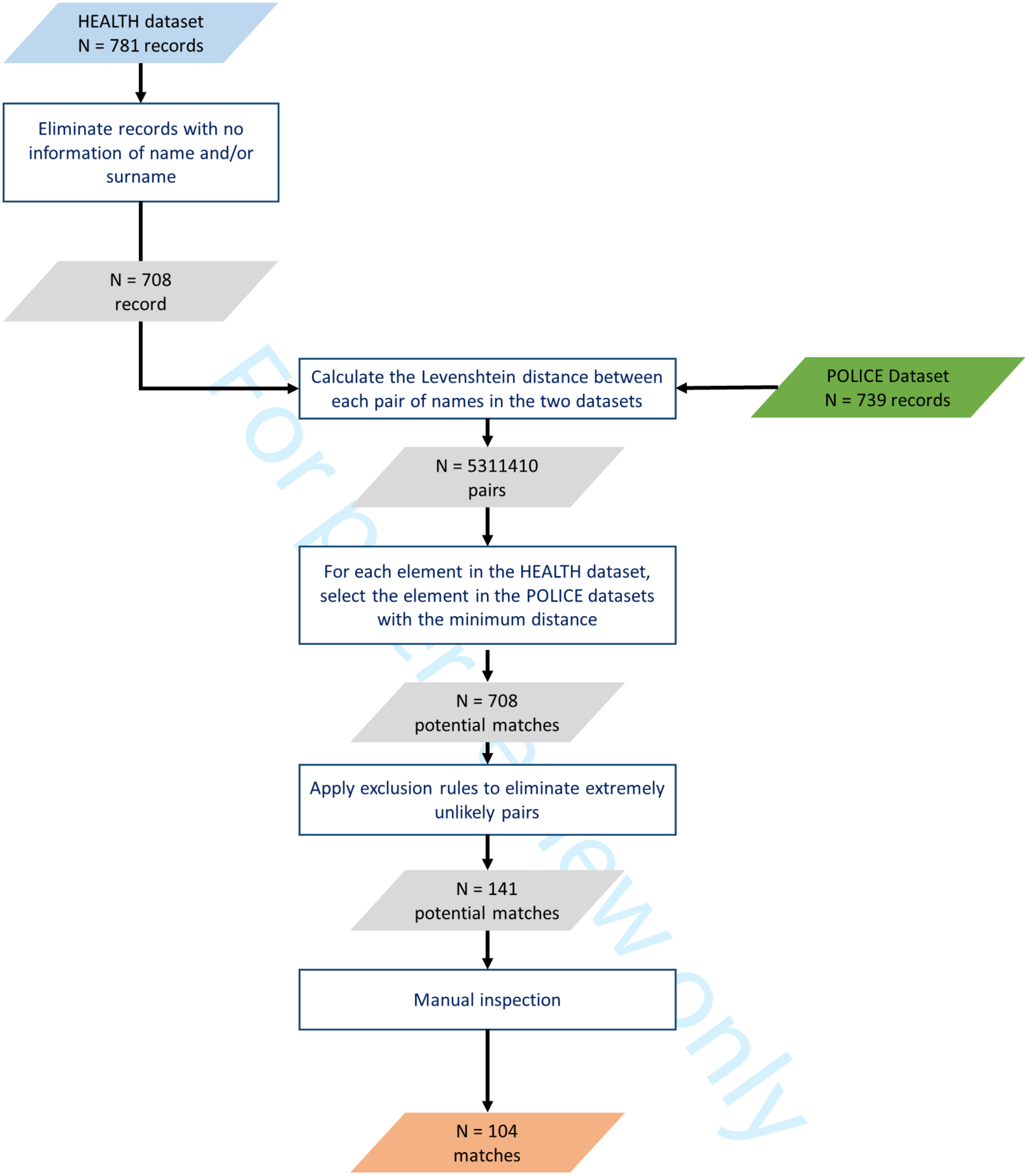

Figure 3. Personal identifier matching between datasets

BMJ Open

MATCHING STUDY USING HEALTH AND POLICE DATASETS FOR CHARECTERISING INTERPERSONAL VIOLENCE IN THE COMMUNITY OF KHAYELITSHA, SOUTH AFRICA 2013-2015

Journal:	*BMJ Open*
Manuscript ID	bmjopen-2020-048129.R1
Article Type:	Original research
Date Submitted by the Author:	30-Aug-2021
Complete List of Authors:	Jabar, Ardil; University of Cape Town, School of Public Health and Family Medicine<br>Oni, Tolu; University of Cape Town, School of Public Health and Family Medicine; University of Cambridge, MRC Epidemiology Unit<br>London, Leslie; University of Cape Town, School of Public Health and Family Medicine<br>Cois, Annibale; Stellenbosch University, Department of Global Health, Division of Health Systems and Public Health, Faculty of Medicine and Health Sciences<br>Matzopoulos, Richard; University of Cape Town, School of Public Health and Family Medicine
**Primary Subject Heading**:	Public health
Secondary Subject Heading:	Epidemiology, Emergency medicine, Evidence based practice, Health informatics
Keywords:	PUBLIC HEALTH, ACCIDENT & EMERGENCY MEDICINE, EPIDEMIOLOGY

SCHOLARONE™
Manuscripts

I, the Submitting Author has the right to grant and does grant on behalf of all authors of the Work (as defined in the below author licence), an exclusive licence and/or a non-exclusive licence for contributions from authors who are: i) UK Crown employees; ii) where BMJ has agreed a CC-BY licence shall apply, and/or iii) in accordance with the terms applicable for US Federal Government officers or employees acting as part of their official duties; on a worldwide, perpetual, irrevocable, royalty-free basis to BMJ Publishing Group Ltd ("BMJ") its licensees and where the relevant Journal is co-owned by BMJ to the co-owners of the Journal, to publish the Work in this journal and any other BMJ products and to exploit all rights, as set out in our licence.

The Submitting Author accepts and understands that any supply made under these terms is made by BMJ to the Submitting Author unless you are acting as an employee on behalf of your employer or a postgraduate student of an affiliated institution which is paying any applicable article publishing charge ("APC") for Open Access articles. Where the Submitting Author wishes to make the Work available on an Open Access basis (and intends to pay the relevant APC), the terms of reuse of such Open Access shall be governed by a Creative Commons licence – details of these licences and which Creative Commons licence will apply to this Work are set out in our licence referred to above.

Other than as permitted in any relevant BMJ Author's Self Archiving Policies, I confirm this Work has not been accepted for publication elsewhere, is not being considered for publication elsewhere and does not duplicate material already published. I confirm all authors consent to publication of this Work and authorise the granting of this licence.

For peer review only

MATCHING STUDY USING HEALTH AND POLICE DATASETS FOR CHARECTERISING INTERPERSONAL VIOLENCE IN THE COMMUNITY OF KHAYELITSHA, SOUTH AFRICA 2013-2015

Ardil Jabar[1], Tolu Oni[2,3], Leslie London[1], Annibale Cois[4], Richard Matzopoulos[1,5]

1. Division of Public Health Medicine, School of Public Health and Family Medicine, University of Cape Town, Cape Town, South Africa
2. Research Initiative for Cities Health and Equity (RICHE), School of Public Health and Family Medicine, University of Cape Town, Cape Town 7925 South Africa
3. MRC Epidemiology unit, University of Cambridge, Cambridge CB2 0QQ, UK
4. Department of Global Health, Division of Health Systems and Public Health, Faculty of Medicine and Health Sciences, Francie van Zijl Drive, Tygerberg, Cape Town
5. Burden of Disease Research Unit, South African Medical Research Council, Tygerberg 7505 Cape Town, South Africa

Corresponding author: Ardil Jabar, a.jabaroo@gmail.com

For peer review only

Abstract

Objectives

The Cardiff Model of data-sharing for violence prevention is premised on the idea that the majority of injury cases presenting at health facilities as a result of interpersonal violence will not be reported to the police. The aim of this study was to determine the concordance between violent crimes reported to the police with violence-related injuries presenting at health facilities in Cape Town, South Africa.

Methods

We conducted a retrospective analysis of secondary cross-sectional health and police data, from three health facilities and three police stations in the community of Khayelitsha, Cape Town. 781 cases of injuries arising from interpersonal violence seen at health facilities were compared with 739 violence-related crimes reported at police stations over five separate week-long sampling periods from 2013 to 2015. Personal identifiers, name and surname, were used to match cases.

Results

Of the 708 cases presenting at health facilities, 104 (14.7%) were matched with police records. The addition of non-reported cases of violence-related injuries from the health dataset to the police reported crime statistics, resulted in an 81.7% increase in potential total violent crimes over the reporting period. Compared to incidents reported to the police, those not reported were more likely to involve male patients (difference: +47.0%; $p<0.001$), and sharp object injuries (difference: +24.7%; $p<0.001$). Push/kick/punch injuries were more frequent among reporting than non-reporting patients (difference: +17.5%; $p<0.001$).

Conclusion

These findings suggest that the majority of injuries arising from interpersonal violence presenting at health facilities in Khayelitsha are not reported to the police. A data sharing model between health services and the police should be implemented to inform violence surveillance and reduction.

For peer review only - http://bmjopen.bmj.com/site/about/guidelines.xhtml

Strengths and limitations of this study

- To the best of our knowledge, this is the first study to attempt direct name matching between the health and the police to quantify the level of underreporting of interpersonal violence-related incidents

- Cross sectional violence-related data was collected from three health facilities and three police precincts within the community of Khayelitsha over a three-year period

- One of the limitations of this study is that patients who presented to a health facility but only lodged criminal cases later outside of the one-week study periods over the three years would not be included in the police dataset and therefore not be included in the matching process.

For peer review only

Introduction

The Cardiff Model employs the strategic use of information from the health sector to improve policing and is an example of cross-sectoral collaboration. Developed by the Violence Research Group at Cardiff University, the model provides a way for communities to gain a clearer picture about where violence occurs by mapping both police and hospital violence data in combination (1). Application of the model in Cardiff in 2011 found that one half to two thirds of violence which results in hospital treatment is not known to the police (2). Subsequent research found that police recording of violence was limited to people reporting violent offenses, but that many of the injured who presented at emergency departments for treatment do not report the incident to police because they are either afraid of reprisals, do not want their own conduct scrutinised, or because they do not think the reporting will result in effective police action (3).

These findings have been replicated in other settings including England and the United States of America (USA), and the model is being considered for implementation in Australia and Jamaica (4)(5). Similarly, findings from the United States (US) Department of Justice National Crime Victimization Survey, a national household survey that sampled 701 000 individuals 12 years and older from 2006 to 2010, revealed that more than half (52%) of all violence incidents in the USA were not reported to law enforcement (6). We were unable to identify any similar studies in South Africa that compared the reporting of violence-related injuries to the police versus health facilities. However, we do know that violence is not always reported to the police. For example, in a community survey in Khayelitsha only 49% of violent incidents between family members were reported to the police (7).

The Western Cape Safety Plan, a policy document developed by the Western Cape Government, advocates the use of data and technology to understand violent crime patterns to inform the deployment of law enforcement resources and investigators accordingly and furthermore acknowledges research and analysis as an important component of its evidence-based policing (EBP) strategy (8). The policy document lends support to the implementation of EBP interventions such as the Cardiff Model locally.

For peer review only - http://bmjopen.bmj.com/site/about/guidelines.xhtml

The aim of this study was to determine the concordance between violent crimes reported to police stations and cases of injuries arising from inter-personal violence presenting to health facilities. Secondary objectives included the estimation of an adjusted crime profile (where police reported crime statistics included unreported violence from the Health data) and a comparison between patient populations that reported violence to the police with those that did not.

Methods

Study setting

Khayelitsha is a peri-urban low-income community of approximately 400 000 people located 30km from the Cape Town city centre (9). The homicide rate is well above the national average of 31 murders per 100 000 population at between 76 and 108 per 100 000 at Khayelitsha's different police stations (10). The community has mixed housing (informal and approximately 45% formal dwellings) and high unemployment (36%) (9) and is serviced by three police stations: Khayelitsha, Harare and Lingelethu West. Trauma cases are treated at three public-sector health facilities: Khayelitsha District Hospital (KDH), Site B and Michael Mapongwana Community Health Centre (Figure 1).

Study design

This was a retrospective cross-sectional analysis of secondary health and crime data from Khayelitsha for five 1-week-long periods collected between September 2013 and October 2015.

We assessed whether people presenting with injuries arising from interpersonal violence at health facilities went on to report these incidents to the police and whether victims reporting a crime to the police sought treatment at a health facility. We quantified this using personal identifier matching.

Data sources

The health data used in this study were originally collected by a non-profit research organisation, the Health Systems Trust (HST), for a study commissioned by the Western Cape Department of Health (DOH). Data were collected for five 1-week-long cross-section periods between 2013 to 2015. The health data were collected via face-to-face interviews with patients with violence-related injuries using a standardised data collection tool loaded on an electronic tablet, in the three health facilities (Figure 1) from 2013 to 2015 (11).

For peer review only - http://bmjopen.bmj.com/site/about/guidelines.xhtml

All emergency cases presenting to the health facilities were assessed for acute injuries which were classified according to cause of injury, viz: violence; transport; other unintentional injury or self-harm. Retrospective folder reviews of sexual assault cases that presented to the clinical forensic units at KDH during the corresponding study period were also included.

The police dataset included all violent crimes reported at all three police stations over the same time periods as the health dataset. This data was recorded in the national South African Police Services (SAPS) Crime Administration System (CAS) in Pretoria. A formal request to access the data was made to the SAPS National Research Division which was granted (Reference no. 3/34/2) on the 24th of November 2017.

For peer review only

Patient and Public Involvement

No patient involved

Variables

Cases presenting to health facilities for violence-related injuries included the following variables: hospital folder number as a personal identifier; age; gender; date and time of injury event; instrument used, type of violence; area (incident location); triage score; and alcohol use.

The inclusion of patient hospital numbers allowed for accessing patients' details on the Clinicom hospital system (a hospital patient tracking information system)(12), to identify patient names for matching with police data.

All acute injuries classified as person-on-person intentional violence were described according to the *external cause of injury* as per the International Classification of Disease (ICD) version 10 (ref), i.e.: sharp object (ICD10 code X99), blunt object (Y00), firearm, push/kick/punch (Y04), human bite (Y08), explosion (X09), choking/strangulation (X91), fire burn (X97), other burn, poisoning (X85), or unknown; and *type of violence* defined in the study as rape/sexual, gang-related, property crime-related, interpersonal or unknown. "Gang-related' and "unknown" were collapsed into a combined 'interpersonal violence' category for purposes of comparison with the police data, which did not include these categories.

Cases recorded in the 'unknown' category included cases that recorded a specific cause of injury, where the type of violence was marked unknown and the victim-perpetrator category was also marked unknown. Patients treated for burns of unknown cause were included in the 'other burn' category.

For peer review only - http://bmjopen.bmj.com/site/about/guidelines.xhtml

The location of each assault was self-reported by patients attending health facilities according to ten predefined study areas (see Figure 1). The collection of incident location data allowed for geolocating each case in one of the ten study areas: Enkanini, Harare, Ilitha Park, Khaya, Kuyasa, Makaya, Makhaza, Site B, Site C, and Town 2 (Figure 1). Cases that were geolocated beyond the ten areas were excluded from the analysis.

The health data also included additional variables of interest that were not available in the police data. The triage colour provided a proxy for injury severity: viz: red (refer to major area for emergency management [immediate]); orange (refer to major area for urgent management [target time < 10 minutes]); yellow (refer to major area for urgent management [target time < 1 hour]); green [refer to designated area for non-urgent management (target time < 4 hours]) and blue (refer to doctor for certification [target time < 2 hours]). Alcohol and other drug use for each case was assessed by clinical judgement. Volume of consumption and consumption beyond six hours of the injury event was not assessed.

The police data included: name and surname as personal identifiers; age; gender; date and time of crime event; type of crime; instrument use; and incident location. Types of crimes included assault with the purpose to inflict grievous bodily harm; attempted murder; common assault; domestic violence; rape; sexual assault; and all types of robbery, which involve an interpersonal interaction with an implicit threat (i.e. common robbery; house robbery; robbery with a weapon or instrument other than a firearm; robbery and attempted robbery with a fire-arm; robbery and attempted robbery at business premises; carjacking [stealing an occupied motor-vehicle]) (13).

For purposes of comparison with the *type of violence* recorded in the health data, crime type was coded as follows: rape and sexual assault cases (rape/sexual); common assault , assault with the purpose to inflict grievous bodily harm, domestic violence attempted murder (interpersonal violence); all types of robbery, which involve an interpersonal interaction with an implicit threat (i.e. common robbery; house robbery; robbery with a weapon or instrument other than a firearm; robbery and attempted robbery with a fire-arm; robbery and attempted robbery at business premises; carjacking [stealing an occupied motor-vehicle] (property crime-related).

The police recorded instrument use as a separate field and the categories were broadly consistent with the health data, as follows: sharp instrument, glass, key, knife, panga (bladed weapon), bottle, bottle head, screwdriver, (sharp object); blunt instrument, hammer, iron pipe, brick, stick, stone/brick (blunt object), firearm, pistol, revolver (firearm), head, fist, body part, feet, hands (push/kick/punch), belt, open hands (choking/strangulation); tyre, matches, petrol bomb (fire burn); electricity, ammunition (other burn); tik [methamphetamine], mandrax pills

For peer review only - http://bmjopen.bmj.com/site/about/guidelines.xhtml

[quaalude], dagga [cannabis] (poisoning); and cell phone, motor vehicle, vehicle, cash, money, bank card, unknown (unknown).

Police incident location data were mapped based on self-reported data provided by victims of violence and were either geolocated using XY co-ordinates or recorded as a street address.

Case selection

Police records with multiple victims were expanded by replicating the common information (place, type of crime, etc.) to generate a single record for each victim in the final dataset.

Cases that were excluded from the health data included medical cases not related to injuries and injuries not caused by violence perpetrated on the victim by another subject (Condition 1); events that occurred outside the ten areas of Khayelitsha (Condition 2); cases in which the victim was younger than 16 years (Condition 3) (age of sexual consent in South Africa); and events that occurred outside the time period specified (Condition 4).

Cases that were removed from the police data include cases other than the types of crimes described above (Condition 1); events that occurred outside the ten areas of Khayelitsha (Condition 2); cases in which the victims were younger than 16 years (Condition 3) and events that occurred outside the time period specified (Condition 4).

Data analysis

The two datasets were captured in Microsoft Excel, and imported into the R Statistical environment v. 3.5.2 for analysis (14). Two-side statistical tests with a conventional 5% cut-off for statistical significance were conducted to assess differences in characteristics between injury patients from the health database and crime victims from the police database, and between reporting and non-reporting patients in the health database.

χ^2 tests were used to evaluate differences in the distribution of categorical variables. When comparing the police dataset with the health dataset, a robust estimator of the test variance was used to take into account the partial overlapping of the two samples (15).

Cases from health facilities were matched with police data using name, surname, gender and age as matching keys. Some latitude was accorded to allow for spelling variations and approximate recording of ages. This was done as follows: patient hospital numbers were entered into the Groote Schuur Hospital (GSH) Clinicom database to retrieve the patient's name. The Levenshtein distance (a string metric for measuring differences between two sequences of characters (16))

For peer review only - http://bmjopen.bmj.com/site/about/guidelines.xhtml

was calculated for each patient between his/her name and each of the names present in the police dataset, and the police record(s) was selected corresponding to the minimum distance as "candidate" matches. In the second step (exclusion of unlikely matches) the potential matches generated from the first step were retained for subsequent analysis if (i) names were identical between the two datasets; or (ii) the Levenshtein distance was 1, gender was the same and reported ages differed less than 5 years; or (iii) the Levenshtein distance was > 1, gender was the same and reported ages differed less than 3 years. In the third step (manual selection) the potential matches resulting from the previous step were manually inspected to verify the actual correspondence between victim names and the consistency of the reported time of the violent episode, which led to the elimination of further cases.

An estimation of an adjusted crime profile was done by adding unreported patient violence seen at health facilities to the police reported crime statistics. The patients who reported violence to police ("reporting patients") and the patients who did not report the violence to the police ("non-reporting patients") were inferred from the matching process, and are described with regards to demographic characteristics (age and gender), instrument use, type of offence, triage code and alcohol use.

Ethics

Ethical permission to conduct this research was granted by the University of Cape Town Human Research Ethics Committee (UCT HREC 861/2016) and the South African Police Services Research Division (3/34/2). With regard to the HST data, informed consent was obtained only from violence-related injury cases and where given, these individuals were interviewed by the data collectors, with only violence-related injury cases having the full data collection tool completed. Informed consent was granted by Groote Schuur Hospital for permission to access the Clinicom patient database in order to complete the personal identifier matching. The study Biostatistician received both health and police datasets, conducted the identifier matching process, and deleted all data permanently following this process. By aggregating the data, removing personal identifiers, geolocating cases to residential suburb and not street address, and employing a one-month delay of data dissemination, every technical effort is made to prevent the identification of individual cases and protect individual identity.

Results

For peer review only - http://bmjopen.bmj.com/site/about/guidelines.xhtml

Summary of included data

The health dataset initially included a total number of 9608 records of patients who presented at the three facilities during the five time periods considered. After applying exclusions (medical conditions, injury not due to interpersonal violence, injury occurring out the study area or study period and child injury), the final dataset included 781 records (Figure 2).

The police dataset initially included a total number of 2161 records of criminal episodes. After applying exclusions (cases occurring outside the five study periods, cases not included in the types of included crimes list, cases where the victim was younger than 16, cases where the incident location was outside one of the ten study areas) the final dataset included 739 records (Figure 2). 358 police records had missing values on gender and age.

Patients seen at health facilities (69.8%) and victims of violence (51.4%) were both more frequently male (Table 1). The mean age for patients was 29.9 for patients and 35.2 for victims of violence. The most common age ranges for both datasets were 25-34 (health 42.2%, police 34.1%), 16-24 (health 33.7%, police 22.3%) and 35-44 (health 15.1%, police 18.9%). At health facilities violence peaked on Sundays (33.7%), whereas violent crimes were most frequently reported to the police on Saturdays (22.3%).

The most common instrument described in the police database was an assault with body part (push/kick/punch) followed by unknown, firearm, sharp object and blunt object. At health facilities, the most common instrument use was sharp objects, followed by an assault with body part (push/kick/punch), blunt objects, unknown and firearms. Sharp objects comprised 60.7% for health presentations versus 12% for police presentations while body part was 41.8% for police station presentation, while only 14.7% for health.

Site B recorded the most cases in both health (36.2%) and police (32.9%) datasets with Ilitha Park (2.7%) and Enkanini (1.2%) recording the fewest cases respectively.

Table 1. Distribution of cases recorded in health facilities and by the police according to gender, age, day of the week, time of day, instrument use, type of violence, incident location, triage score and alcohol/drug use

Variable	HEALTH data (N=781)	POLICE data (N=739)
	n (%)	n (%)

For peer review only - http://bmjopen.bmj.com/site/about/guidelines.xhtml

Name Surname completeness	708 (90.7)	739 (100)
**Gender***		
Males	**545 (69.8)**	**196 (51.4)**
Females	**236 (30.2)**	**185 (48.6)**
**Age (years)***		
16-24	263 (33.7)	85 (22.3)
25-34	330 (42.2)	130 (34.1)
35-44	118 (15.1)	72 (18.9)
45-54	46 (5.9)	56 (14.7)
55+	24 (3.1)	38 (10.0)
**Day of week**		
Monday	95 (12.2)	103 (13.9)
Tuesday	50 (6.4)	72 (9.7)
Wednesday	48 (6.1)	75 (10.1)
Thursday	45 (5.8)	64 (8.7)
Friday	77 (9.8)	100 (13.5)
Saturday	203 (26.0)	165 (22.3)
Sunday	263 (33.7)	160 (21.7)
**Time of day**		
00h00:07h59	212 (27.1)	202 (27.3)
08h00:15h59	252 (32.3)	196 (26.5)
16h00:23h59	317 (40.5)	341 (46.1)
**Instrument used**		
Sharp object	474 (60.7)	89 (12.0)
Blunt object	112 (14.3)	70 (9.5)
Firearm	29 (3.7)	89 (12.0)
Push/kick/punch	115 (14.7)	309 (41.8)
Human bite	6 (0.8)	2 (0.3)
Choking/strangulation	2 (0.3)	8 (1.1)
Fire burn	2 (0.3)	5 (0.7)
Other burn	3 (0.4)	2 (0.3)
Poisoning	0 (0.0)	56 (7.6)
Unknown	38 (4.9)	109 (14.7)
**Type of violence**		

For peer review only - http://bmjopen.bmj.com/site/about/guidelines.xhtml

Rape/sexual	16 (2.0)	67 (9.1)
Property crime-related	290 (37.0)	250 (33.8)
Interpersonal	266 (44.8)	422 (57.1)
Gang-related	84 (10.7)	
Unknown	125 (16.0)	**
**Area**		
Enkanini	41 (5.2)	9 (1.2)
Harare	85 (10.9)	66 (8.9)
Ilitha Park	21 (2.7)	42 (5.7)
Khaya	30 (3.8)	74 (10.0)
Kuyasa	39 (5.0)	45 (6.1)
Makaya	43 (5.5)	37 (5.0)
Makhaza	85 (11.0)	96 (13.0)
Site B	283 (36.2)	243 (32.9)
Site C	118 (15.1)	91(12.3)
Town 2	36 (4.6)	36 (4.9)
**Triage scores** ***		
Red	65 (8.3%)	-
Orange	251 (32.1%)	-
Yellow	380 (48.6%)	-
Green	68 (8.7%)	-
Blue	1 (0.12)	-
Unknown	18 (2.2%)	-
**Alcohol use** ***		
Yes	444 (56.8%)	-
No	284 (36.4%)	-
Unknown	53 (6.8%)	-

* 358 police records had missing values on gender and age

** For type of violence, the police data did not record an 'unknown or gang-related' category

*** Triage score and alcohol use data were only available for the health dataset

For peer review only - http://bmjopen.bmj.com/site/about/guidelines.xhtml

For peer review only

For peer review only

Concordance

Among the 708 patients being treated for violence-related injuries at health facilities for which the name and surname could be retrieved from the Clinicom database, only 104 reported the incident to the police, which equates to a matching ratio of 14.7% (Figure 3).

Adjusted crime profile

Combining unreported cases from health records to the police reported crime statistics provides an adjusted crime profile (Table 2). The addition of patient violence from the health data to the police reported crime statistics, resulted in an 81.7% increase in potential total violent crimes over the reporting period. Males represent the majority in the combined (65.0%) dataset. In terms of day of week, Saturday and Sunday were the busiest days in the combined dataset with Thursday being the quietest.

Push/kick/punch (41.8% and 40.3%) is the most common instrument used in the police and combined dataset respectively, with the commonest type of violence being interpersonal violence for both (57.1 and 58.1%). Site B, Site C and Makhaza ranked amongst the top three for both datasets with Enkanini (1.5%) similarly recording the fewest cases.

For peer review only - http://bmjopen.bmj.com/site/about/guidelines.xhtml

Notable shifts in ranking when combining the datasets were found in day of week (Saturday ranked 1 in police versus 2 in combined dataset), time of day, instrument used (push/kick/punch ranked 1 in police data [41.8%], and sharp object ranked 1 in combined data [35.4%]), and area where the positions for Makhaza and Site C were changed in the top 3 ranked areas.

For peer review

Table 2. Adjusted crime profile comparing police versus police and health combined data

Variable	POLICE data (N=739)		Combined Data (N=1343)	
	Rank	n (%)	Rank	n (%)
**Gender***				
Males	1	196 (51.4)	1	640 (65.0)
Females	2	185 (48.6)	2	345 (35.0)
**Age (years)***				
16-24	2	85 (22.3)	2	274 (27.8)
25-34	1	130 (34.1)	1	394 (40.0)
35-44	3	72 (18.9)	3	173 (17.6)
45-54	4	56 (14.7)	4	87 (8.8)
55+	5	38 (10.0)	5	57 (5.8)
**Day of week**				
Monday	3	103 (13.9)	3	178 (13.2)
Tuesday	6	72 (9.7)	6	107 (8.0)
Wednesday	5	75 (10.1)	5	109 (8.1)
Thursday	7	64 (8.7)	7	96 (7.1)
Friday	4	100 (13.5)	4	155 (11.5)
Saturday	1	165 (22.3)	2	320 (23.8)

For peer review only - http://bmjopen.bmj.com/site/about/guidelines.xhtml

Sunday	2	160 (21.7)	1	378 (28.1)
**Time of day**				
00h00:07h59	2	202 (27.3)	3	362 (26.9)
08h00:15h59	3	196 (26.5)	2	390 (29)
16h00:23h59	1	341 (46.1)	1	591 (43.9)
**Instrument used**				
Sharp object	3	89 (12.0)	1	476 (35.4)
Blunt object	4	70 (9.5)	5	155 (11.5)
Firearm	3	89 (12.0)	4	111 (8.3)
Push/kick/punch	1	309 (41.8)	2	383 (28.5)
Human bite	8	2 (0.3)	9	5 (0.4)
Choking/strangulation	6	8 (1.1)	7	9 (0.7)
Fire burn	7	5 (0.7)	8	7 (0.5)
Other burn	8	2 (0.3)	10	5 (0.4)
Poisoning	5	56 (7.6)	6	56 (4.2)
Unknown	2	109 (14.7)	3	136 (10.1)
**Type of violence**				
Rape/sexual	3	67 (9.1)	3	74 (5.5)
Property crime-related	2	250 (33.8)	2	489 (36.4)
Interpersonal **	1	422 (57.1)	1	780 (58.1)
**Area**				
Enkanini	10	9 (1.2)	8	41 (3.1)
Harare	5	66 (8.9)	4	129 (9.6)
Ilitha Park	7	42 (5.7)	7	59 (4.4)
Khaya	4	74 (10.0)	4	96 (7.1)
Kuyasa	6	45 (6.1)	5	74 (5.5)
Makaya	8	37 (5.0)	6	70 (5.2)
Makhaza	2	96 (13.0)	3	163 (12.1)
Site B	1	243 (32.9)	1	461 (34.3)
Site C	3	91(12.3)	2	188 (14.0)
Town 2	9	36 (4.9)	7	62 (4.6)

*358 police records had missing values on gender and age

For peer review only - http://bmjopen.bmj.com/site/about/guidelines.xhtml

** For purposes of comparison, 'gang-related' cases from the health data were included as 'interpersonal violence' cases. Additionally, the 'unknown 'category was included as 'interpersonal violence' for purposes of comparison and ranking.

Predictors of non-reporting

Male patients (73.5%) that were stabbed (64.1%), had alcohol use confirmed or suspected (58.9%) and experienced crime (39.6%) or gang-related incidents (11.4%) were less likely to report their injuries to the police. Patients suffering sharp object (39.4%) or push/kick/punch injuries (29.8%) or sexual assault/rape (8.7%) were more likely to report their injuries (Table 3).

No statistically significant differences were present regarding age and severity of the injury (as measured by the triage code). There were minimal differences in location and timing of the violent episode between reporting and non-reporting patients. The one significant difference in timing of the violence episode was the almost double number of episodes reported on Sunday among non-reporting patients when compared to reporting patients (36.1% vs. 20.2%, p=0.017).

Table 3. Difference in demographic and clinical characteristics and in type of offence between patients who did/did not report the violence to police

**Variable**		Patients who **did not report** the violence: N = 604	Patients who **reported** the violence: N = 104	**All** patients: * N = 708	
		**n (%)**	**n (%)**	**n (%)**	**p-value****
**Gender**					
	Male	444 (73.5%)	44 (42.3%)	488 (68.9%)	
	Female	160 (26.5%)	60 (57.7%)	220 (31.1%)	**<0.001**
**Age (years)**					
	16-24	188 (31.2)	41 (39.4)	229 (32.4)	
	25-34	264 (43.8)	34 (32.7)	298 (42.1)	**0.017**
	35-44	101 (16.7)	16 (15.4)	117 (16.5)	
	45-54	31 (5.1)	12 (11.5)	43 (6.1)	

For peer review only - http://bmjopen.bmj.com/site/about/guidelines.xhtml

55+	20 (3.2)	1 (1.0)	21 (2.8)	
**Day of week**				
Monday	75 (12.4)	14 (13.5)	89 (12.6)	
Tuesday	35 (5.8)	12 (11.5)	47 (6.6)	
Wednesday	34 (5.6)	10 (9.6)	44 (6.2)	
Thursday	32 (5.3)	5 (4.8)	37 (5.2)	**0.017**
Friday	55 (9.1)	15 (14.4)	70 (9.9)	
Saturday	155 (25.7)	27 (26)	182 (25.7)	
Sunday	218 (36.1)	21 (20.2)	239 (33.8)	
**Time of day**				
00h00:07h59	160 (26.4)	30 (28.9)	190 (26.8)	
08h00:15h59	194 (32.2)	39 (37.4)	233 (33)	0.267
16h00:23h59	250 (41.4)	35 (33.6)	285 (40.3)	
**Instrument**				
Sharp object	387 (64.1)	41 (39.4)	428 (60.5)	
Blunt object	85 (14.1)	18 (17.3)	103 (14.5)	
Firearm	22 (3.6)	5 (4.8)	27 (3.8)	
Push/kick/punch	74 (12.3)	31 (29.8)	105 (14.8)	**<0.001**
Human bite	3 (0.5)	1 (1)	4 (0.6)	
Choking/strangulation	1 (0.2)	1 (1)	2 (0.3)	
Burns	5 (0.8)	0 (0)	5 (0.7)	
Unknown	27 (4.5)	7 (6.7)	34 (4.8)	
**Type of violence**				
Rape/sexual	7 (1.2)	9 (8.7)	16 (2.3)	
Property crime-related***	239 (39.6)	26 (25.0)	265 (37.4)	
Interpersonal	199 (33.0)	45 (43.2)	244 (34.5)	**<0.001**
Gang-related	69 (11.4)	6 (5.8)	75 (10.6)	
Unknown	90 (14.9)	18 (17.3)	108 (15.3)	
**Area**				
Enkanini	32 (5.3)	4 (3.8)	36 (5.1)	
Harare	63 (10.4)	14 (13.5)	77 (10.9)	
Ilitha Park	17 (2.8)	1 (1)	18 (2.5)	
Khaya	22 (3.6)	4 (3.8)	26 (3.7)	0.689
Kuyasa	29 (4.8)	6 (5.8)	35 (4.9)	
Makaya	33 (5.5)	7 (6.7)	40 (5.6)	
Makhaza	67 (11.1)	13 (12.5)	80 (11.3)	

For peer review only - http://bmjopen.bmj.com/site/about/guidelines.xhtml

Site B	218 (36.1)	38 (36.5)	256 (36.2)	
Site C	97 (16.1)	10 (9.6)	107 (15.1)	
Town 2	26 (4.3)	7 (6.7)	33 (4.7)	
**Triage code**				
Red	47 (7.8)	7 (6.7)	54 (7.6)	
Orange	204 (33.8)	23 (22.1)	227 (32.1)	0.278
Yellow	290 (48)	58 (55.8)	348 (49.2)	
Green	53 (8.8)	8 (7.7)	61 (8.6)	
Blue	1 (0.2)	0 (0)	1 (0.1)	
Unknown	9 (1.5)	8 (7.7)	17 (2.4)	
**Alcohol use**				
Yes/Suspected	356 (58.9)	49 (47.1)	405 (57.2)	
No	205 (33.9)	50 (48.1)	255 (36)	**0.012**
Unknown	43 (7.1)	5 (4.8)	48 (6.8)	

* For which the name and surname could be retrieved from the Clinicom database;
** Statistical test for the existence of differences between reporting and not-reporting patients.
*** Property crime-related refers to the type of violence that could not be categorised under rape/sexual, interpersonal violence, gang violence or unknown. Cases in this category included violence initiated to facilitate property theft from an individual.

Discussion

One of the key findings of a 2014 systematic review of the feasibility and effectiveness of community-level interventions involving emergency department data sharing to reduce alcohol-related violence, was that data sharing protocols can be cheaply and easily implemented into modern Emergency Department (ED) triage systems (17). There was general consensus among studies that this could be done with minimal cost, staff workload burden and impact to patient safety. Additionally, there was minimum risk to patient service and anonymity, risk of harm displacement to other licensed venues or increase to length of patient stay (17). The Cardiff Model is cost-effective with one study finding that for every $1 spent, nearly $15 in health system and over $19 in criminal justice system costs are saved (18).

To the best of our knowledge, this is the first study to attempt direct name matching between the health and the police to quantify the level of underreporting of interpersonal violence-related incidents. The matching method using personal identifiers suggest that only a small

For peer review only

fraction (approximately 15 %) of patients being treated for interpersonal violence at local health facilities will go on to report these as crimes to local police stations within a week. Conversely, only a small proportion of those who report a violent crime to the police go on to present to a health facility with an injury within a week.

The proportion of cases in this study of injuries arising from interpersonal violence which are not reported to the police (approximately 82%), is consistent with the findings of the Cardiff model which found that one half to two thirds of violence which results in hospital treatment is not known to the police (2). This finding is much lower when compared with other regions including in developed countries. According to a 2016 U.S. Department of Justice report, many crimes go unreported to law enforcement, including 53% of violent crime in 2015; 58% of simple assaults in 2015 and 43% of violent crime involving an injury in 2015 (19).

Possible reasons for the high level of underreporting (30%+ discrepancy between the level of under reporting in this study [82%) and the Cardiff model [50%]), can be found in the report of the 2012 Khayelitsha Commission of inquiry (also known as the O'Regan/Pikoli Commission) which investigated allegations of police inefficiency in Khayelitsha and the breakdown in relations between the Khayelitsha community and the police. Poor policing conditions characterised by police in the area being generally lower-ranked, poorly equipped, few in number, underqualified and reluctant to open cases, led to breakdown in trust with the community resulting in the lack of reporting (20). Other possible reasons for underreporting include the fear of reprisal from perpetrators, being turned away by police when presenting at the police station to report a crime, stigma/shame, or not perceiving an injury arising from interpersonal violence as a crime.

Type of injury

The majority of police reported crimes are related to assault with body part (push/kick/punch). Victims of knife crimes represented in the health data, which often cause worse injuries requiring medical intervention, are not reporting these incidents to police. Gang/Property crime-related episodes were more common among non-reporting patients, which may be due to a lack of confidence in the police's ability to protect them from reprisal, or if the victim perceived the gangs as being protected by or working with the police, in a known context of mistrust of police in the community of Khayelitsha (20). Additionally, the underreporting of knife wielding gang-related violence incidents in police statistics may be a contributing factor. This is supported by the lower proportion of younger male victims (age 16-24) reporting crime compared to those receiving treatment for an injury arising from interpersonal violence (31.2% vs 39.4%, P=0.017).

For peer review only - http://bmjopen.bmj.com/site/about/guidelines.xhtml

The scale in difference of sharp object injuries presenting at police (89 cases) and health facilities (474), means that 385 victims of violence treated for a sharp object injury at a health facility did not go on to report the crime to the police, which if added to the official police statistics would represent a 52% increase in all violent crimes reported. This provides evidence for an excess burden of injuries arising from interpersonal violence which is not being reported to police – a situation that is consistent with the premise of the Cardiff model.

Limitations

One of the limitations of this study is that approximately 10% of health records lacked names and were thus excluded from the matching process. A 2013 South African study in missing medical records found that up to 8% of hospital folders had the wrong names and that 17% of hospital folders had names without folder numbers (21).

No cases of poisoning were recorded in the health data. The current referral practice is for Michael M and Site B to refer their poisoning cases to KDH. At KDH most poisoning cases are triaged to the Medical department and thus these cases would not be recorded in the health data. The exception being cases of poisoning due to a corrosive substance such as bleach, which would be triaged to the Trauma/Surgical department due to the possible need for a surgical therapeutic intervention. It is a recommendation of study authors that the poisoning data from medical emergencies be included to inform this work going forward.

One of the weaknesses of this study is that patients who presented to a health facility but only lodged criminal cases later outside of the one-week study periods over the five years would not be included in the police dataset and therefore not be included in the matching process. Similarly, if victims of injuries arising from interpersonal violence presented to the police first and did not present to the health facilities for treatment within the one-week study period, they were also not included in the matching process. The inclusion of these late reporting cases could decrease the overall proportion of interpersonal violence-related injuries that were reported to the police. Finally, patients experiencing inter-personal violence and who sought private health care or presented directly to the hospitals outside of the district (such as the tertiary referral hospital, Tygerberg Hospital) would not be included in the analyses. This may increase or decrease the estimate of concordance depending on whether these patients reported to the police. **Implications for policy and interventions**

Murder and violence are often linked to alcohol with a 2016 analysis of murder dockets finding that in up to 48% of murders, the victim or perpetrator was intoxicated (22). Alcohol use was higher in patients who did not report violence than patients who did report violence to the police. This is of public health importance as alcohol has been identified as a major risk factor

For peer review only - http://bmjopen.bmj.com/site/about/guidelines.xhtml

for and predictor of violence and is amenable to policy change to reduce alcohol harms. The current evidence base for measuring alcohol use within crime data will underestimate the extent of the problem because of the underreporting in routine crime statistics highlighted by this research.

Furthermore, with regard to local municipal alcohol policy on liquor outlet licences (23), reasons for limitation of liquor outlet licences may include, 'safety and social problems and/or concerns arising from alcohol abuse, alcohol related crimes, illegal criminal activity, etc, using data derived from the South African Police Service'. The local alcohol policy is heavily reliant on the SAPS data to guide license approvals, which this research has shown to be incomplete and flawed in its underestimation of interpersonal violence at the community level. Alcohol policy decisions made by Government at any level (local, district, provincial and national) that is informed by this data, may aggravate alcohol related harms.

The level of underreporting exposed by the merging of health and police datasets and matching exercise may speak to a larger question of police resourcing within Khayelitsha. The Khayelitsha commission of inquiry highlighted the insufficient police human resources within police stations in Khayelitsha as one of the reasons for community mistrust of the police(20). The current SAPS resource allocation model is based on the Theoretical Human Resource Requirement (THRR) and fixed establishment (FE) approach which was developed in 2012 (24) . The tool takes into consideration annual reported crime per police station (24) which suggests that current police human resources deployed in Khayelitsha may be severely under resourced to deal with the 'real' burden of violence, when unreported health data is considered.

The validity of self-reported drinking using ED data from 16 countries reported findings suggesting that the overall validity of self-reported alcohol consumption before injury is high (88%) compared with a blood alcohol concentration (BAC) estimate obtained at the time of admission to the ED, with a caveat that demographic, drinking and injury characteristics may affect validity(25).

This study provides evidence showing that interpersonal violence at the community level cannot be estimated accurately relying solely on reported crimes to the police services. Injuries arising from interpersonal violence treated at health facilities must be included to more accurately define the level of interpersonal violence within a community.

Implementation of the Hospital Emergency Centre Tracking Information System (HECTIS), an electronic data collection system for Emergency Departments (ED), is currently underway at the Michael Mapongwana CHC and KDH. This will improve the monitoring of injuries arising from interpersonal violence (the majority of which are not reported to the police) and provide a

For peer review only - http://bmjopen.bmj.com/site/about/guidelines.xhtml

method for the evaluation of violence prevention interventions. Additionally, this will provide the data infrastructure required for the potential implementation of the Cardiff model locally.

Preliminary findings from this research were shared with the Whole of Society Approach (WOSA) intergovernmental task team that operates in the community of Khayelitsha, with a mandate to reduce interpersonal violence. The full findings will be shared with the WOSA stakeholders as preliminary work to inform the full implementation of the Cardiff model in Khayelitsha early next year.

Conclusion

The matching exercise described above represents the first attempt within South Africa to match patients at health facilities treated for injuries arising from interpersonal violence with those of victims reporting violent crimes to police stations within the same community and time period. This matching study suggests that the majority of injuries arising from interpersonal violence presenting at health facilities in Khayelitsha are not reported to the police in Khayelitsha.

This study has broader implications regionally and nationally for the surveillance of injuries arising from interpersonal violence, for the police definition and surveillance of community interpersonal violence, for community policing intelligence development (improving the configuration of violence heat maps on a real time basis) and finally for police resource utilisation and distribution, which should, in turn, impact positively on reducing crime and violence in the community, and reduce the burden on the Health services. The implementation of the Cardiff data sharing model should be adopted to inform violence reduction locally.

Ethics

Ethical permission to conduct this research was granted by the University of Cape Town Human Research Ethics Committee (UCT HREC 861/2016) and the South African Police Services Research Division (Application no. 3/34/2). With regard to the HST data, informed consent was obtained only from acute injury cases and where given, these individuals were interviewed by the data collectors, with only acute injury cases having the full data collection tool completed. Informed consent was granted by Groote Schuur Hospital for permission to access the Clinicom patient database. No minors were interviewed as part of this study.

There are no competing interests for any author.

For peer review only

AJ and RM developed the research proposal and were involved in the analysis and interpretation of the data. AC was involved with the analysis of the data including the matching protocol. LL and TO revised the work for content and approved the final version to be published.

There is no additional data available.

Funding

This work was supported by the International Development Research Centre (IDRC), Canada, and the UK Government's Department for International Development (DFID) (Grant number 107329–001) and the Western Cape Government Department of Health (Grant number WCDOH428/2013). The degree from which this study emanated was funded by the South African Medical Research Council (SAMRC) under the SAMRC Clinician Researcher (MD PhD) Scholarship Programme. RM is funded by the SAMRC. TO is supported by the National Institute for Health Research (NIHR) (16/137/34) using UK aid from the UK Government to support global health research. The views expressed in this publication are those of the authors and not necessarily those of the NIHR or the UK Department of Health and Social Care or SAMRC.

Figure 1. Participating health facilities and police stations in relation to study areas in the community of Khayelitsha

Figure 2. Case selection flow chart for health and police datasets

Figure 3. Personal identifier matching between datasets

References

1. CDC. Community Guidance for Violence Prevention Cardiff Model Toolkit [Internet]. Atlanta; 2018. Available from: https://www.cdc.gov/violenceprevention/fundedprograms/cardiffmodel/toolkit.html

2. Florence C, Shepherd J, Brennan I, Simon T. Effectiveness of anonymised information sharing and use in health service, police, and local government partnership for preventing violence related injury: experimental study and time series analysis. BMJ. 2011;342:d3313.

3. Group violence research. Cardiff Model for Violence Prevention Implementation of the Model in the UK International [Internet]. Vol. 44. Cardiff; 2018. Available from: https://static1.squarespace.com/static/57875c16197aea2902e3820e/t/5a03139f71c10ba51be806ee/1510151075166/vrg+cardiff+model+briefing+web.pdf

4. Kollar L, Sumner S, Bartholow B, Wu D, Moore J, Mays E, et al. Building capacity for injury

For peer review only - http://bmjopen.bmj.com/site/about/guidelines.xhtml

prevention : a process evaluation of a replication of the Cardiff Violence Prevention Programme in the Southeastern USA. Inj Prev. 2019;1–8.

5. Boyle A a, Snelling K, White L, Ariel B, Ashelford L. External validation of the Cardiff model of information sharing to reduce community violence: natural experiment. Emerg Med J [Internet]. 2013;30(12):1020–3. Available from: http://www.ncbi.nlm.nih.gov/pubmed/23184923

6. Langton L, Berzofsky M, Krebs C, Smily-McDonald H. Victimizations not reported to police [Internet]. Bureau of Justice statistics. [cited 2017 Feb 17]. Available from: http://www.bjs.gov/content/pub/pdf/vnrp0610.pdf.2012

7. Cassidy T, Lloyd S, Bowman B, Myers J, Parry C, Makanga T, et al. Authors : [Internet]. Cape Town; 2013. Available from: https://open.uct.ac.za/bitstream/handle/11427/28271/Cassidy_Survey violence alcohol.pdf?sequence=3

8. Government WC. Western Cape Safety Plan Working Document. Cape Town; 2019.

9. City of Cape Town. City of Cape Town – 2011 Census Suburb Khayelitsha [Internet]. Cape Town; 2011. Available from: https://www.capetown.gov.za/en/stats/2011CensusSuburbs/2011_Census_CT_Suburb_Khayelitsha_Profile.pdf

10. Barolsky V. Violence in Khayelitsha: finding a way out. Cape Town; 2014.

11. Mureithi L, Africa A, Wandai M, Schaik N Van, Naledi T, Matzopoulos R, et al. Injury Morbidity Surveillance in Nyanga and Khayelitsha in the Western Cape. Vol. 27. Cape Town; 2016.

12. Government WC. New Patient Administrative (AR) and Billing system at Prince Albert Hospital [Internet]. Western Cape Government news. 2020 [cited 2020 Nov 17]. Available from: https://www.westerncape.gov.za/news/new-patient-administrative-ar-and-billing-system-prince-albert-hospital

13. SAPS. SAPS crime defintions. SAPS Website. 2019.

14. R Core Team. R: A language and environment for statistical computing [Internet]. R Foundation for Statistical Computing. 2013 [cited 2020 Sep 3]. Available from: http://www.r-project.org/

15. Zeileis A. Object-Oriented Computation of Sandwich Estimators. J Stat Softw. 2006;16(9):1–16.

16. Doan A, Halevy A, Ives Z. Principles of Data Integration. 1st ed. Morgan Kaufmann; 2012. 95–119 p.

17. Droste N, Miller P, Baker T. Review article: Emergency department data sharing to

For peer review only - http://bmjopen.bmj.com/site/about/guidelines.xhtml

reduce alcohol-related violence: A systematic review of the feasibility and effectiveness of community-level interventions. Emerg Med Australas [Internet]. 2014;26(4):326–35. Available from: http://doi.wiley.com/10.1111/1742-6723.12247

18. Holder, Yvette et al. Injury Surveillance Guidelines. Geneva; 2001.

19. Morgan R, Kena G. Criminal Victimization,2016:Revised [Internet]. Bureau of Justice Statistics. 2018. Available from: https://www.bjs.gov/content/pub/pdf/cv16.pdf

20. WCG. Report of the Commission of Inquiry into Allegations of Police Inefficiency and a Breakdown in Relations between SAPS and the Community of Khayelitsha. Cape Town; 2014.

21. Wegner L, Rhoda A. Missing medical records : an obstacle to archival survey-research in a rural community in South Africa. South African J Physiother. 2013;15–9.

22. SAPS. Crime statistics South Africa [Internet]. Cape Town; 2016. Available from: https://www.saps.gov.za/services/crimestats.php

23. Municipality D. Drakenstein Municipality Liquor Outlet and Tavern draft Policy [Internet]. Cape Town: Drakenstein Municipality; 2020. Available from: http://www.drakenstein.gov.za/docs/Documents/DM Liquor Policy - 3rd draft_20200723.pdf

24. Government WC. Analyses of Human Resource Allocation for South African Police Service with specific reference to the Western Cape. Cape Town; 2017.

25. Cherpitel C, Ye Y, Bond J, G B, Macdonald S, Stockwell T, et al. Validity of self-reported drinking before injury compared with a physiological measure: cross national analysis of Emergency department data from 16 countries. 2006.

For peer review only

For peer review only - http://bmjopen.bmj.com/site/about/guidelines.xhtml

Figure 1

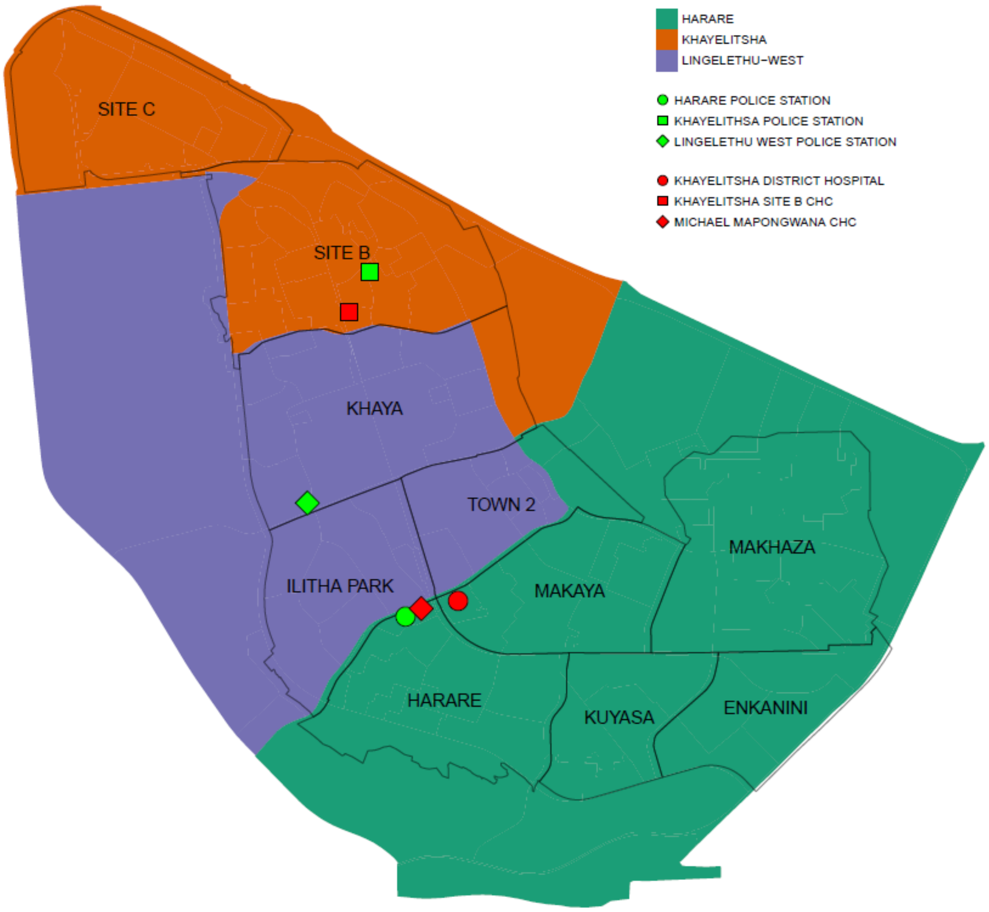

Figure 1. Participating health facilities and police stations in relation to study areas in the community of Khayelitsha

Figure 2

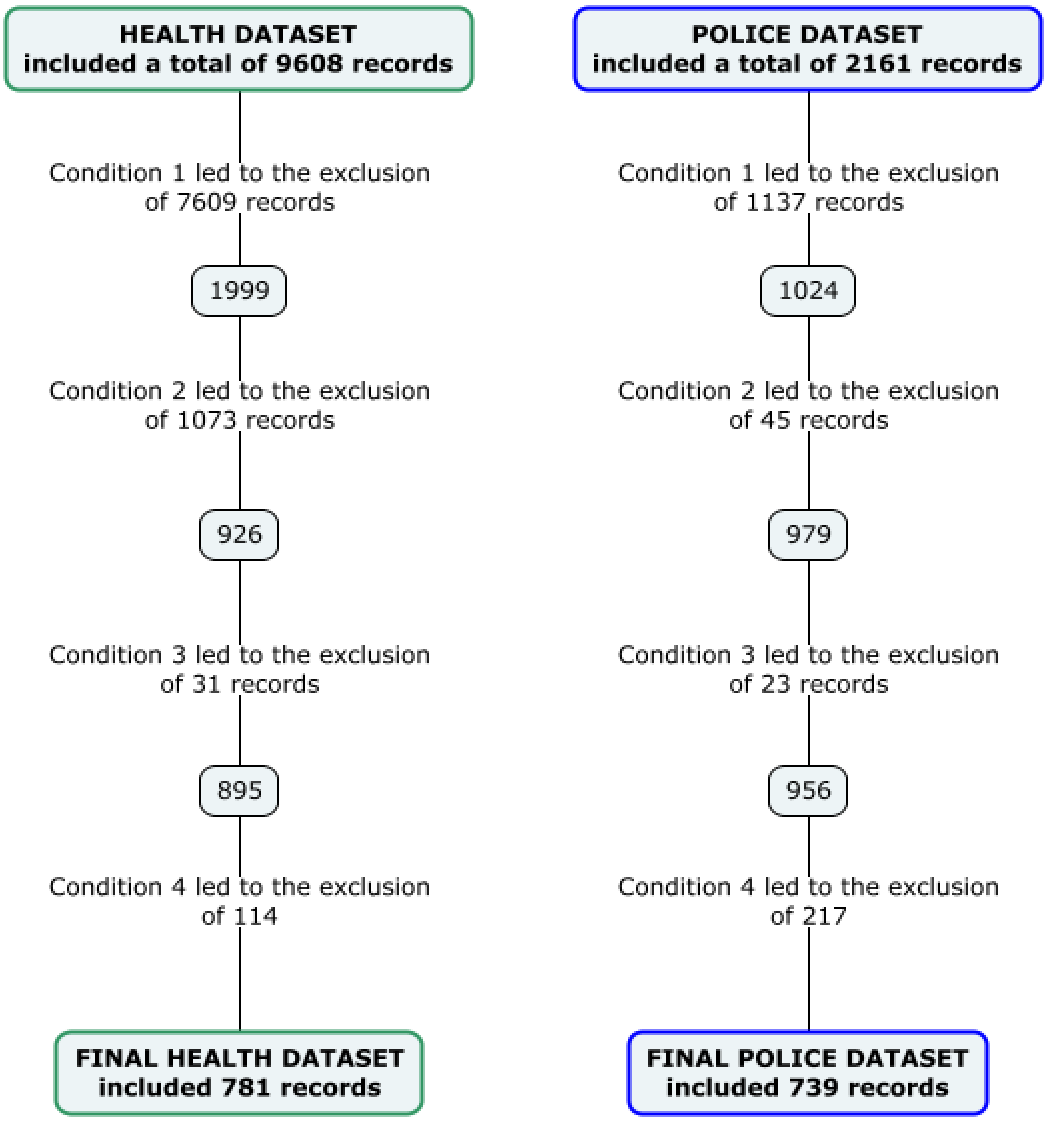

KEY:

Health condition 1: Cases that were excluded from the health data included medical cases not related to injuries and injuries not caused by violence perpetrated on the victim by another subject

Police condition 1: Cases that were removed from the police data include cases other than the types of crimes described in variables section

Condition 2: events that occurred outside the ten areas of Khayelitsha

Condition 3: cases in which the victim was younger than 16 years

Condition 4: events that occurred outside the time period

Figure 2. Case selection flow chart for health and police datasets

For peer review only - http://bmjopen.bmj.com/site/about/guidelines.xhtml

Figure 3

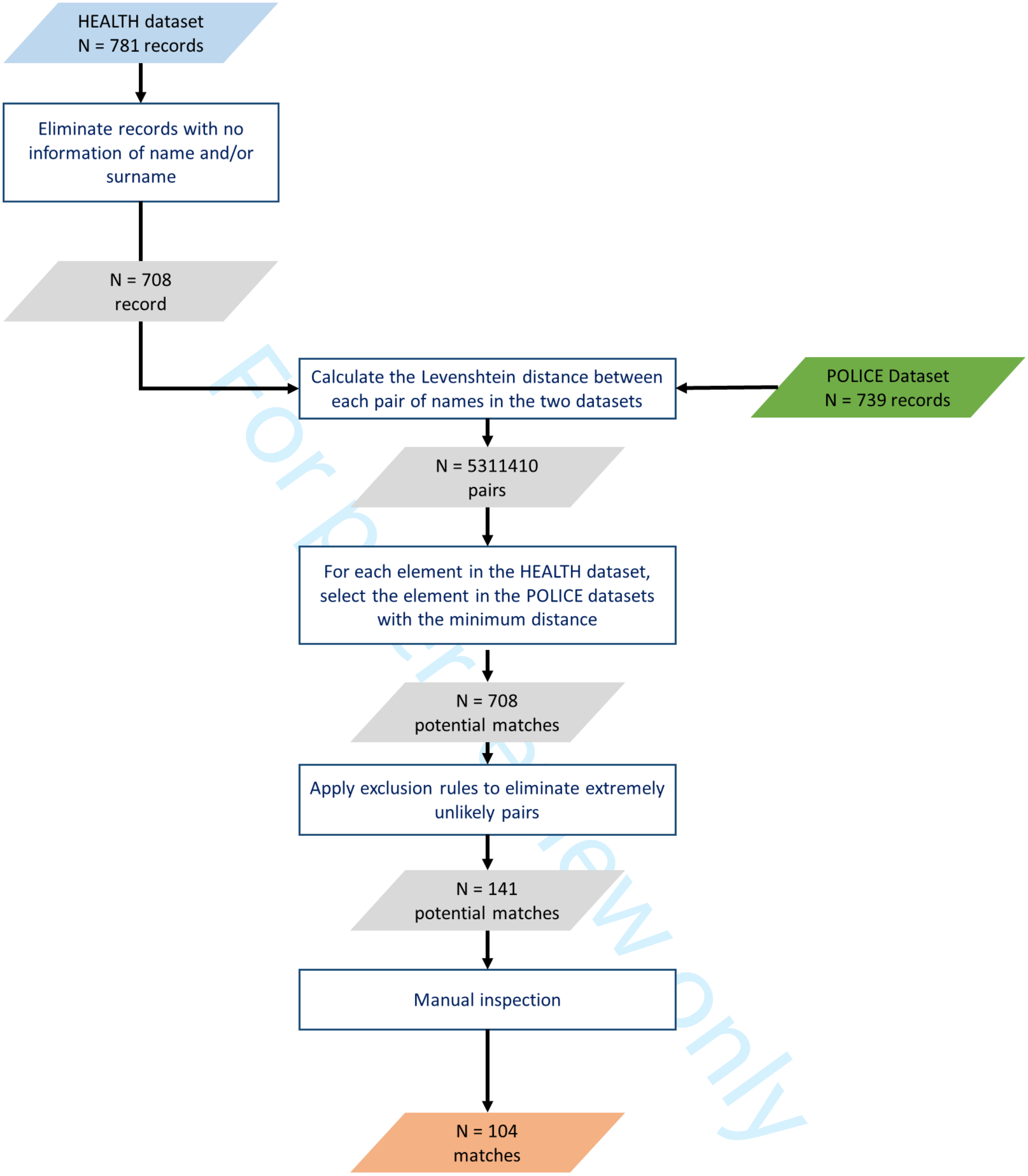

Figure 3. Personal identifier matching between datasets

BMJ Open

MATCHING STUDY USING HEALTH AND POLICE DATASETS FOR CHARACTERISING INTERPERSONAL VIOLENCE IN THE COMMUNITY OF KHAYELITSHA, SOUTH AFRICA 2013-2015

Journal:	*BMJ Open*
Manuscript ID	bmjopen-2020-048129.R2
Article Type:	Original research
Date Submitted by the Author:	02-Jun-2022

Complete List of Authors:	Jabar, Ardil; University of Cape Town, School of Public Health and Family Medicine<br>Oni, Tolu; University of Cape Town, School of Public Health and Family Medicine; University of Cambridge, MRC Epidemiology Unit<br>London, Leslie; University of Cape Town, School of Public Health and Family Medicine<br>Cois, Annibale; Stellenbosch University, Department of Global Health, Division of Health Systems and Public Health, Faculty of Medicine and Health Sciences<br>Matzopoulos, Richard; University of Cape Town, School of Public Health and Family Medicine

**Primary Subject Heading**:	Public health
Secondary Subject Heading:	Epidemiology, Emergency medicine, Evidence based practice, Health informatics
Keywords:	PUBLIC HEALTH, ACCIDENT & EMERGENCY MEDICINE, EPIDEMIOLOGY

SCHOLARONE™
Manuscripts

BMJ

I, the Submitting Author has the right to grant and does grant on behalf of all authors of the Work (as defined in the below author licence), an exclusive licence and/or a non-exclusive licence for contributions from authors who are: i) UK Crown employees; ii) where BMJ has agreed a CC-BY licence shall apply, and/or iii) in accordance with the terms applicable for US Federal Government officers or employees acting as part of their official duties; on a worldwide, perpetual, irrevocable, royalty-free basis to BMJ Publishing Group Ltd ("BMJ") its licensees and where the relevant Journal is co-owned by BMJ to the co-owners of the Journal, to publish the Work in this journal and any other BMJ products and to exploit all rights, as set out in our licence.

The Submitting Author accepts and understands that any supply made under these terms is made by BMJ to the Submitting Author unless you are acting as an employee on behalf of your employer or a postgraduate student of an affiliated institution which is paying any applicable article publishing charge ("APC") for Open Access articles. Where the Submitting Author wishes to make the Work available on an Open Access basis (and intends to pay the relevant APC), the terms of reuse of such Open Access shall be governed by a Creative Commons licence – details of these licences and which Creative Commons licence will apply to this Work are set out in our licence referred to above.

Other than as permitted in any relevant BMJ Author's Self Archiving Policies, I confirm this Work has not been accepted for publication elsewhere, is not being considered for publication elsewhere and does not duplicate material already published. I confirm all authors consent to publication of this Work and authorise the granting of this licence.

For peer review only

MATCHING STUDY USING HEALTH AND POLICE DATASETS FOR CHARACTERISING INTERPERSONAL VIOLENCE IN THE COMMUNITY OF KHAYELITSHA, SOUTH AFRICA 2013-2015

Ardil Jabar[1], Tolu Oni[2,3], Leslie London[1], Annibale Cois[4], Richard Matzopoulos[1,5]

1. Division of Public Health Medicine, School of Public Health and Family Medicine, University of Cape Town, Cape Town, South Africa
2. Research Initiative for Cities Health and Equity (RICHE), School of Public Health and Family Medicine, University of Cape Town, Cape Town 7925 South Africa
3. MRC Epidemiology unit, University of Cambridge, Cambridge CB2 0QQ, UK
4. Department of Global Health, Division of Health Systems and Public Health, Faculty of Medicine and Health Sciences, Francie van Zijl Drive, Tygerberg, Cape Town
5. Burden of Disease Research Unit, South African Medical Research Council, Tygerberg 7505 Cape Town, South Africa

Corresponding author: Ardil Jabar, a.jabaroo@gmail.com

For peer review only - http://bmjopen.bmj.com/site/about/guidelines.xhtml

Abstract

Objectives

The Cardiff Model of data-sharing for violence prevention is premised on the idea that the majority of injury cases presenting at health facilities as a result of interpersonal violence will not be reported to the police. The aim of this study was to determine the concordance between violent crimes reported to the police with violence-related injuries presenting at health facilities in Cape Town, South Africa.

Methods

We conducted a retrospective analysis of secondary cross-sectional health and police data, from three health facilities and three police stations in the community of Khayelitsha, Cape Town. 781 cases of injuries arising from interpersonal violence seen at health facilities were compared with 739 violence-related crimes reported at police stations over five separate week-long sampling periods from 2013 to 2015. Personal identifiers, name and surname, were used to match cases.

Results

Of the 708 cases presenting at health facilities, 104 (14.7%) were matched with police records. The addition of non-reported cases of violence-related injuries from the health dataset to the police reported crime statistics, resulted in an 81.7% increase in potential total violent crimes over the reporting period. Compared to incidents reported to the police, those not reported were more likely to involve male patients (difference: +47.0%; $p<0.001$), and sharp object injuries (difference: +24.7%; $p<0.001$). Push/kick/punch injuries were more frequent among reporting than non-reporting patients (difference: +17.5%; $p<0.001$).

Conclusion

These findings suggest that the majority of injuries arising from interpersonal violence presenting at health facilities in Khayelitsha are not reported to the police. A data sharing model between health services and the police should be implemented to inform violence surveillance and reduction.

For peer review only - http://bmjopen.bmj.com/site/about/guidelines.xhtml

Strengths and limitations of this study

- To the best of our knowledge, this is the first study to attempt direct name matching between the health and the police to quantify the level of underreporting of interpersonal violence-related incidents
- Cross sectional violence-related data was collected from three health facilities and three police precincts within the community of Khayelitsha over a three-year period
- One of the limitations of this study is that patients who presented to a health facility but only lodged criminal cases later outside of the one-week study periods over the three years would not be included in the police dataset and therefore not be included in the matching process.

For peer review only

Introduction

The Cardiff Model employs the strategic use of information from the health sector to improve policing and is an example of cross-sectoral collaboration. Developed by the Violence Research Group at Cardiff University, the model provides a way for communities to gain a clearer picture about where violence occurs by mapping both police and hospital violence data in combination (1). Application of the model in Cardiff in 2011 found that one half to two thirds of violence which results in hospital treatment is not known to the police (2). Subsequent research found that police recording of violence was limited to people reporting violent offenses, but that many of the injured who presented at emergency departments for treatment do not report the incident to police because they are either afraid of reprisals, do not want their own conduct scrutinised, or because they do not think the reporting will result in effective police action (3).

These findings have been replicated in other settings including England and the United States of America (USA), and the model is being considered for implementation in Australia and Jamaica (4)(5). Similarly, findings from the United States (US) Department of Justice National Crime Victimization Survey, a national household survey that sampled 701 000 individuals 12 years and older from 2006 to 2010, revealed that more than half (52%) of all violence incidents in the USA were not reported to law enforcement (6). We were unable to identify any similar studies in South Africa that compared the reporting of violence-related injuries to the police versus health facilities. However, we do know that violence is not always reported to the police. For example, in a community survey in Khayelitsha only 49% of violent incidents between family members were reported to the police (7).

The Western Cape Safety Plan, a policy document developed by the Western Cape Government, advocates the use of data and technology to understand violent crime patterns to inform the deployment of law enforcement resources and investigators accordingly and furthermore acknowledges research and analysis as an important component of its evidence-based policing (EBP) strategy (8). The policy document lends support to the implementation of EBP interventions such as the Cardiff Model locally.

For peer review only - http://bmjopen.bmj.com/site/about/guidelines.xhtml

The aim of this study was to determine the concordance between violent crimes reported to police stations and cases of injuries arising from inter-personal violence presenting to health facilities. Secondary objectives included the estimation of an adjusted crime profile (where police reported crime statistics included unreported violence from the Health data) and a comparison between patient populations that reported violence to the police with those that did not.

Methods

Study setting

Khayelitsha is a peri-urban low-income community of approximately 400 000 people located 30km from the Cape Town city centre (9). The homicide rate is well above the national average of 31 murders per 100 000 population at between 76 and 108 per 100 000 at Khayelitsha's different police stations (10). The community has mixed housing (informal and approximately 45% formal dwellings) and high unemployment (36%) (9) and is serviced by three police stations: Khayelitsha, Harare and Lingelethu West. Trauma cases are treated at three public-sector health facilities: Khayelitsha District Hospital (KDH), Site B and Michael Mapongwana Community Health Centre (Figure 1).

Study design

This was a retrospective cross-sectional analysis of secondary health and crime data from Khayelitsha for five 1-week-long periods collected between September 2013 and October 2015.

We assessed whether people presenting with injuries arising from interpersonal violence at health facilities went on to report these incidents to the police and whether victims reporting a crime to the police sought treatment at a health facility. We quantified this using personal identifier matching.

Data sources

The health data used in this study were originally collected by a non-profit research organisation, the Health Systems Trust (HST), for a study commissioned by the Western Cape Department of Health (DOH). Data were collected for five 1-week-long cross-section periods between 2013 to 2015. The health data were collected via face-to-face interviews with patients with violence-related injuries using a standardised data collection tool loaded on an electronic tablet, in the three health facilities (Figure 1) from 2013 to 2015 (11).

For peer review only - http://bmjopen.bmj.com/site/about/guidelines.xhtml

All emergency cases presenting to the health facilities were assessed for acute injuries which were classified according to cause of injury, viz: violence; transport; other unintentional injury or self-harm. Retrospective folder reviews of sexual assault cases that presented to the clinical forensic units at KDH during the corresponding study period were also included.

The police dataset included all violent crimes reported at all three police stations over the same time periods as the health dataset. This data was recorded in the national South African Police Services (SAPS) Crime Administration System (CAS) in Pretoria. A formal request to access the data was made to the SAPS National Research Division which was granted (Reference no. 3/34/2) on the 24th of November 2017.

Patient and Public Involvement

No patient involved

Variables

Cases presenting to health facilities for violence-related injuries included the following variables: hospital folder number as a personal identifier; age; gender; date and time of injury event; instrument used, type of violence; area (incident location); triage score; and alcohol use.

The inclusion of patient hospital numbers allowed for accessing patients' details on the Clinicom hospital system (a hospital patient tracking information system)(12), to identify patient names for matching with police data.

All acute injuries classified as person-on-person intentional violence were described according to the *external cause of injury* as per the International Classification of Disease (ICD) version 10 (ref), i.e.: sharp object (ICD10 code X99), blunt object (Y00), firearm, push/kick/punch (Y04), human bite (Y08), explosion (X09), choking/strangulation (X91), fire burn (X97), other burn, poisoning (X85), or unknown; and *type of violence* defined in the study as rape/sexual, gang-related, property crime-related, interpersonal or unknown. "Gang-related' and "unknown" were collapsed into a combined 'interpersonal violence' category for purposes of comparison with the police data, which did not include these categories.

Cases recorded in the 'unknown' category included cases that recorded a specific cause of injury, where the type of violence was marked unknown and the victim-perpetrator category was also marked unknown. Patients treated for burns of unknown cause were included in the 'other burn' category.

For peer review only - http://bmjopen.bmj.com/site/about/guidelines.xhtml

The location of each assault was self-reported by patients attending health facilities according to ten predefined study areas (see Figure 1). The collection of incident location data allowed for geolocating each case in one of the ten study areas: Enkanini, Harare, Ilitha Park, Khaya, Kuyasa, Makaya, Makhaza, Site B, Site C, and Town 2 (Figure 1). Cases that were geolocated beyond the ten areas were excluded from the analysis.

The health data also included additional variables of interest that were not available in the police data. The triage colour provided a proxy for injury severity: viz: red (refer to major area for emergency management [immediate]); orange (refer to major area for urgent management [target time < 10 minutes]); yellow (refer to major area for urgent management [target time < 1 hour]); green [refer to designated area for non-urgent management (target time < 4 hours]) and blue (refer to doctor for certification [target time < 2 hours]). Alcohol and other drug use for each case was assessed by clinical judgement. Volume of consumption and consumption beyond six hours of the injury event was not assessed.

The police data included: name and surname as personal identifiers; age; gender; date and time of crime event; type of crime; instrument use; and incident location. Types of crimes included assault with the purpose to inflict grievous bodily harm; attempted murder; common assault; domestic violence; rape; sexual assault; and all types of robbery, which involve an interpersonal interaction with an implicit threat (i.e. common robbery; house robbery; robbery with a weapon or instrument other than a firearm; robbery and attempted robbery with a fire-arm; robbery and attempted robbery at business premises; carjacking [stealing an occupied motor-vehicle]) (13).

For purposes of comparison with the *type of violence* recorded in the health data, crime type was coded as follows: rape and sexual assault cases (rape/sexual); common assault , assault with the purpose to inflict grievous bodily harm, domestic violence attempted murder (interpersonal violence); all types of robbery, which involve an interpersonal interaction with an implicit threat (i.e. common robbery; house robbery; robbery with a weapon or instrument other than a firearm; robbery and attempted robbery with a fire-arm; robbery and attempted robbery at business premises; carjacking [stealing an occupied motor-vehicle] (property crime-related).

The police recorded instrument use as a separate field and the categories were broadly consistent with the health data, as follows: sharp instrument, glass, key, knife, panga (bladed weapon), bottle, bottle head, screwdriver, (sharp object); blunt instrument, hammer, iron pipe, brick, stick, stone/brick (blunt object), firearm, pistol, revolver (firearm), head, fist, body part, feet, hands (push/kick/punch), belt, open hands (choking/strangulation); tyre, matches, petrol bomb (fire burn); electricity, ammunition (other burn); tik [methamphetamine], mandrax pills

For peer review only - http://bmjopen.bmj.com/site/about/guidelines.xhtml

[quaalude], dagga [cannabis] (poisoning); and cell phone, motor vehicle, vehicle, cash, money, bank card, unknown (unknown).

Police incident location data were mapped based on self-reported data provided by victims of violence and were either geolocated using XY co-ordinates or recorded as a street address.

Case selection

Police records with multiple victims were expanded by replicating the common information (place, type of crime, etc.) to generate a single record for each victim in the final dataset.

Cases that were excluded from the health data included medical cases not related to injuries and injuries not caused by violence perpetrated on the victim by another subject (Condition 1); events that occurred outside the ten areas of Khayelitsha (Condition 2); cases in which the victim was younger than 16 years (Condition 3) (age of sexual consent in South Africa); and events that occurred outside the time period specified (Condition 4).

Cases that were removed from the police data include cases other than the types of crimes described above (Condition 1); events that occurred outside the ten areas of Khayelitsha (Condition 2); cases in which the victims were younger than 16 years (Condition 3) and events that occurred outside the time period specified (Condition 4).

Data analysis

The two datasets were captured in Microsoft Excel, and imported into the R Statistical environment v. 3.5.2 for analysis (14). Two-side statistical tests with a conventional 5% cut-off for statistical significance were conducted to assess differences in characteristics between injury patients from the health database and crime victims from the police database, and between reporting and non-reporting patients in the health database.

χ^2 tests were used to evaluate differences in the distribution of categorical variables. When comparing the police dataset with the health dataset, a robust estimator of the test variance was used to take into account the partial overlapping of the two samples (15).

Cases from health facilities were matched with police data using name, surname, gender and age as matching keys. Some latitude was accorded to allow for spelling variations and approximate recording of ages. This was done as follows: patient hospital numbers were entered into the Groote Schuur Hospital (GSH) Clinicom database to retrieve the patient's name. The Levenshtein distance (a string metric for measuring differences between two sequences of characters (16))

For peer review only - http://bmjopen.bmj.com/site/about/guidelines.xhtml

was calculated for each patient between his/her name and each of the names present in the police dataset, and the police record(s) was selected corresponding to the minimum distance as "candidate" matches. In the second step (exclusion of unlikely matches) the potential matches generated from the first step were retained for subsequent analysis if (i) names were identical between the two datasets; or (ii) the Levenshtein distance was 1, gender was the same and reported ages differed less than 5 years; or (iii) the Levenshtein distance was > 1, gender was the same and reported ages differed less than 3 years. In the third step (manual selection) the potential matches resulting from the previous step were manually inspected to verify the actual correspondence between victim names and the consistency of the reported time of the violent episode, which led to the elimination of further cases.

An estimation of an adjusted crime profile was done by adding unreported patient violence seen at health facilities to the police reported crime statistics. The patients who reported violence to police ("reporting patients") and the patients who did not report the violence to the police ("non-reporting patients") were inferred from the matching process, and are described with regards to demographic characteristics (age and gender), instrument use, type of offence, triage code and alcohol use.

Ethics

Ethical permission to conduct this research was granted by the University of Cape Town Human Research Ethics Committee (UCT HREC 861/2016) and the South African Police Services Research Division (3/34/2). With regard to the HST data, informed consent was obtained only from violence-related injury cases and where given, these individuals were interviewed by the data collectors, with only violence-related injury cases having the full data collection tool completed. Informed consent was granted by Groote Schuur Hospital for permission to access the Clinicom patient database in order to complete the personal identifier matching. The study Biostatistician received both health and police datasets, conducted the identifier matching process, and deleted all data permanently following this process. By aggregating the data, removing personal identifiers, geolocating cases to residential suburb and not street address, and employing a one-month delay of data dissemination, every technical effort is made to prevent the identification of individual cases and protect individual identity.

Results

For peer review only - http://bmjopen.bmj.com/site/about/guidelines.xhtml

Summary of included data

The health dataset initially included a total number of 9608 records of patients who presented at the three facilities during the five time periods considered. After applying exclusions (medical conditions, injury not due to interpersonal violence, injury occurring out the study area or study period and child injury), the final dataset included 781 records (Figure 2).

The police dataset initially included a total number of 2161 records of criminal episodes. After applying exclusions (cases occurring outside the five study periods, cases not included in the types of included crimes list, cases where the victim was younger than 16, cases where the incident location was outside one of the ten study areas) the final dataset included 739 records (Figure 2). 358 police records had missing values on gender and age.

Patients seen at health facilities (69.8%) and victims of violence (51.4%) were both more frequently male (Table 1). The mean age for patients was 29.9 for patients and 35.2 for victims of violence. The most common age ranges for both datasets were 25-34 (health 42.2%, police 34.1%), 16-24 (health 33.7%, police 22.3%) and 35-44 (health 15.1%, police 18.9%). At health facilities violence peaked on Sundays (33.7%), whereas violent crimes were most frequently reported to the police on Saturdays (22.3%).

The most common instrument described in the police database was an assault with body part (push/kick/punch) followed by unknown, firearm, sharp object and blunt object. At health facilities, the most common instrument use was sharp objects, followed by an assault with body part (push/kick/punch), blunt objects, unknown and firearms. Sharp objects comprised 60.7% for health presentations versus 12% for police presentations while body part was 41.8% for police station presentation, while only 14.7% for health.

Site B recorded the most cases in both health (36.2%) and police (32.9%) datasets with Ilitha Park (2.7%) and Enkanini (1.2%) recording the fewest cases respectively.

Table 1. Distribution of cases recorded in health facilities and by the police according to gender, age, day of the week, time of day, instrument use, type of violence, incident location, triage score and alcohol/drug use

Variable	HEALTH data (N=781)	POLICE data (N=739)
	n (%)	n (%)

**Name Surname completeness**	**708 (90.7)**	**739 (100)**
**Gender***		
Males	**545 (69.8)**	**196 (51.4)**
Females	**236 (30.2)**	**185 (48.6)**
**Age (years)***		
16-24	263 (33.7)	85 (22.3)
25-34	330 (42.2)	130 (34.1)
35-44	118 (15.1)	72 (18.9)
45-54	46 (5.9)	56 (14.7)
55+	24 (3.1)	38 (10.0)
**Day of week**		
Monday	95 (12.2)	103 (13.9)
Tuesday	50 (6.4)	72 (9.7)
Wednesday	48 (6.1)	75 (10.1)
Thursday	45 (5.8)	64 (8.7)
Friday	77 (9.8)	100 (13.5)
Saturday	203 (26.0)	165 (22.3)
Sunday	263 (33.7)	160 (21.7)
**Time of day**		
00h00:07h59	212 (27.1)	202 (27.3)
08h00:15h59	252 (32.3)	196 (26.5)
16h00:23h59	317 (40.5)	341 (46.1)
**Instrument used**		
Sharp object	474 (60.7)	89 (12.0)
Blunt object	112 (14.3)	70 (9.5)
Firearm	29 (3.7)	89 (12.0)
Push/kick/punch	115 (14.7)	309 (41.8)
Human bite	6 (0.8)	2 (0.3)
Choking/strangulation	2 (0.3)	8 (1.1)
Fire burn	2 (0.3)	5 (0.7)
Other burn	3 (0.4)	2 (0.3)
Poisoning	0 (0.0)	56 (7.6)
Unknown	38 (4.9)	109 (14.7)
**Type of violence**		

For peer review only - http://bmjopen.bmj.com/site/about/guidelines.xhtml

Rape/sexual	16 (2.0)	67 (9.1)
Property crime-related	290 (37.0)	250 (33.8)
Interpersonal	266 (44.8)	422 (57.1)
Gang-related	84 (10.7)	
Unknown	125 (16.0)	**
**Area**		
Enkanini	41 (5.2)	9 (1.2)
Harare	85 (10.9)	66 (8.9)
Ilitha Park	21 (2.7)	42 (5.7)
Khaya	30 (3.8)	74 (10.0)
Kuyasa	39 (5.0)	45 (6.1)
Makaya	43 (5.5)	37 (5.0)
Makhaza	85 (11.0)	96 (13.0)
Site B	283 (36.2)	243 (32.9)
Site C	118 (15.1)	91(12.3)
Town 2	36 (4.6)	36 (4.9)
**Triage scores** ***		
Red	65 (8.3%)	-
Orange	251 (32.1%)	-
Yellow	380 (48.6%)	-
Green	68 (8.7%)	-
Blue	1 (0.12)	-
Unknown	18 (2.2%)	-
**Alcohol use** ***		
Yes	444 (56.8%)	-
No	284 (36.4%)	-
Unknown	53 (6.8%)	-

* 358 police records had missing values on gender and age

** For type of violence, the police data did not record an ‘unknown or gang-related’ category

*** Triage score and alcohol use data were only available for the health dataset

For peer review only - http://bmjopen.bmj.com/site/about/guidelines.xhtml

Concordance

Among the 708 patients being treated for violence-related injuries at health facilities for which the name and surname could be retrieved from the Clinicom database, only 104 reported the incident to the police, which equates to a matching ratio of 14.7% (Figure 3).

Adjusted crime profile

Combining unreported cases from health records to the police reported crime statistics provides an adjusted crime profile (Table 2). The addition of patient violence from the health data to the police reported crime statistics, resulted in an 81.7% increase in potential total violent crimes over the reporting period. Males represent the majority in the combined (65.0%) dataset. In terms of day of week, Saturday and Sunday were the busiest days in the combined dataset with Thursday being the quietest.

Push/kick/punch (41.8% and 40.3%) is the most common instrument used in the police and combined dataset respectively, with the commonest type of violence being interpersonal violence for both (57.1 and 58.1%). Site B, Site C and Makhaza ranked amongst the top three for both datasets with Enkanini (1.5%) similarly recording the fewest cases.

For peer review only - http://bmjopen.bmj.com/site/about/guidelines.xhtml

Notable shifts in ranking when combining the datasets were found in day of week (Saturday ranked 1 in police versus 2 in combined dataset), time of day, instrument used (push/kick/punch ranked 1 in police data [41.8%], and sharp object ranked 1 in combined data [35.4%]), and area where the positions for Makhaza and Site C were changed in the top 3 ranked areas.

For peer review only

Table 2. Adjusted crime profile comparing police versus police and health combined data

Variable	POLICE data (N=739)		Combined Data (N=1343)	
	Rank	n (%)	Rank	n (%)
**Gender***				
Males	1	196 (51.4)	1	640 (65.0)
Females	2	185 (48.6)	2	345 (35.0)
**Age (years)***				
16-24	2	85 (22.3)	2	274 (27.8)
25-34	1	130 (34.1)	1	394 (40.0)
35-44	3	72 (18.9)	3	173 (17.6)
45-54	4	56 (14.7)	4	87 (8.8)
55+	5	38 (10.0)	5	57 (5.8)
**Day of week**				
Monday	3	103 (13.9)	3	178 (13.2)
Tuesday	6	72 (9.7)	6	107 (8.0)
Wednesday	5	75 (10.1)	5	109 (8.1)
Thursday	7	64 (8.7)	7	96 (7.1)
Friday	4	100 (13.5)	4	155 (11.5)
Saturday	1	165 (22.3)	2	320 (23.8)

For peer review only - http://bmjopen.bmj.com/site/about/guidelines.xhtml

Sunday	2	160 (21.7)	1	378 (28.1)
**Time of day**				
00h00:07h59	2	202 (27.3)	3	362 (26.9)
08h00:15h59	3	196 (26.5)	2	390 (29)
16h00:23h59	1	341 (46.1)	1	591 (43.9)
**Instrument used**				
Sharp object	3	89 (12.0)	1	476 (35.4)
Blunt object	4	70 (9.5)	5	155 (11.5)
Firearm	3	89 (12.0)	4	111 (8.3)
Push/kick/punch	1	309 (41.8)	2	383 (28.5)
Human bite	8	2 (0.3)	9	5 (0.4)
Choking/strangulation	6	8 (1.1)	7	9 (0.7)
Fire burn	7	5 (0.7)	8	7 (0.5)
Other burn	8	2 (0.3)	10	5 (0.4)
Poisoning	5	56 (7.6)	6	56 (4.2)
Unknown	2	109 (14.7)	3	136 (10.1)
**Type of violence**				
Rape/sexual	3	67 (9.1)	3	74 (5.5)
Property crime-related	2	250 (33.8)	2	489 (36.4)
Interpersonal **	1	422 (57.1)	1	780 (58.1)
**Area**				
Enkanini	10	9 (1.2)	8	41 (3.1)
Harare	5	66 (8.9)	4	129 (9.6)
Ilitha Park	7	42 (5.7)	7	59 (4.4)
Khaya	4	74 (10.0)	4	96 (7.1)
Kuyasa	6	45 (6.1)	5	74 (5.5)
Makaya	8	37 (5.0)	6	70 (5.2)
Makhaza	2	96 (13.0)	3	163 (12.1)
Site B	1	243 (32.9)	1	461 (34.3)
Site C	3	91(12.3)	2	188 (14.0)
Town 2	9	36 (4.9)	7	62 (4.6)

*358 police records had missing values on gender and age

For peer review only - http://bmjopen.bmj.com/site/about/guidelines.xhtml

** For purposes of comparison, ‘gang-related’ cases from the health data were included as ‘interpersonal violence’ cases. Additionally, the ‘unknown ‘category was included as ‘interpersonal violence’ for purposes of comparison and ranking.

Predictors of non-reporting

Male patients (73.5%) that were stabbed (64.1%), had alcohol use confirmed or suspected (58.9%) and experienced crime (39.6%) or gang-related incidents (11.4%) were less likely to report their injuries to the police. Patients suffering sharp object (39.4%) or push/kick/punch injuries (29.8%) or sexual assault/rape (8.7%) were more likely to report their injuries (Table 3).

No statistically significant differences were present regarding age and severity of the injury (as measured by the triage code). There were minimal differences in location and timing of the violent episode between reporting and non-reporting patients. The one significant difference in timing of the violence episode was the almost double number of episodes reported on Sunday among non-reporting patients when compared to reporting patients (36.1% vs. 20.2%, p=0.017).

Table 3. Difference in demographic and clinical characteristics and in type of offence between patients who did/did not report the violence to police

**Variable**	Patients who **did not report** the violence: N = 604	Patients who **reported** the violence: N = 104	**All** patients: * N = 708	
	**n (%)**	**n (%)**	**n (%)**	**p-value****
**Gender**				
Male	444 (73.5%)	44 (42.3%)	488 (68.9%)	
Female	160 (26.5%)	60 (57.7%)	220 (31.1%)	**<0.001**
**Age (years)**				
16-24	188 (31.2)	41 (39.4)	229 (32.4)	
25-34	264 (43.8)	34 (32.7)	298 (42.1)	**0.017**
35-44	101 (16.7)	16 (15.4)	117 (16.5)	
45-54	31 (5.1)	12 (11.5)	43 (6.1)	

For peer review only - http://bmjopen.bmj.com/site/about/guidelines.xhtml

55+	20 (3.2)	1 (1.0)	21 (2.8)	
**Day of week**				
Monday	75 (12.4)	14 (13.5)	89 (12.6)	
Tuesday	35 (5.8)	12 (11.5)	47 (6.6)	
Wednesday	34 (5.6)	10 (9.6)	44 (6.2)	
Thursday	32 (5.3)	5 (4.8)	37 (5.2)	**0.017**
Friday	55 (9.1)	15 (14.4)	70 (9.9)	
Saturday	155 (25.7)	27 (26)	182 (25.7)	
Sunday	218 (36.1)	21 (20.2)	239 (33.8)	
**Time of day**				
00h00:07h59	160 (26.4)	30 (28.9)	190 (26.8)	
08h00:15h59	194 (32.2)	39 (37.4)	233 (33)	0.267
16h00:23h59	250 (41.4)	35 (33.6)	285 (40.3)	
**Instrument**				
Sharp object	387 (64.1)	41 (39.4)	428 (60.5)	
Blunt object	85 (14.1)	18 (17.3)	103 (14.5)	
Firearm	22 (3.6)	5 (4.8)	27 (3.8)	
Push/kick/punch	74 (12.3)	31 (29.8)	105 (14.8)	**<0.001**
Human bite	3 (0.5)	1 (1)	4 (0.6)	
Choking/strangulation	1 (0.2)	1 (1)	2 (0.3)	
Burns	5 (0.8)	0 (0)	5 (0.7)	
Unknown	27 (4.5)	7 (6.7)	34 (4.8)	
**Type of violence**				
Rape/sexual	7 (1.2)	9 (8.7)	16 (2.3)	
Property crime-related***	239 (39.6)	26 (25.0)	265 (37.4)	
Interpersonal	199 (33.0)	45 (43.2)	244 (34.5)	**<0.001**
Gang-related	69 (11.4)	6 (5.8)	75 (10.6)	
Unknown	90 (14.9)	18 (17.3)	108 (15.3)	
**Area**				
Enkanini	32 (5.3)	4 (3.8)	36 (5.1)	
Harare	63 (10.4)	14 (13.5)	77 (10.9)	
Ilitha Park	17 (2.8)	1 (1)	18 (2.5)	
Khaya	22 (3.6)	4 (3.8)	26 (3.7)	0.689
Kuyasa	29 (4.8)	6 (5.8)	35 (4.9)	
Makaya	33 (5.5)	7 (6.7)	40 (5.6)	
Makhaza	67 (11.1)	13 (12.5)	80 (11.3)	

For peer review only - http://bmjopen.bmj.com/site/about/guidelines.xhtml

Site B	218 (36.1)	38 (36.5)	256 (36.2)	
Site C	97 (16.1)	10 (9.6)	107 (15.1)	
Town 2	26 (4.3)	7 (6.7)	33 (4.7)	
**Triage code**				
Red	47 (7.8)	7 (6.7)	54 (7.6)	
Orange	204 (33.8)	23 (22.1)	227 (32.1)	0.278
Yellow	290 (48)	58 (55.8)	348 (49.2)	
Green	53 (8.8)	8 (7.7)	61 (8.6)	
Blue	1 (0.2)	0 (0)	1 (0.1)	
Unknown	9 (1.5)	8 (7.7)	17 (2.4)	
**Alcohol use**				
Yes/Suspected	356 (58.9)	49 (47.1)	405 (57.2)	
No	205 (33.9)	50 (48.1)	255 (36)	**0.012**
Unknown	43 (7.1)	5 (4.8)	48 (6.8)	

* For which the name and surname could be retrieved from the Clinicom database;
** Statistical test for the existence of differences between reporting and not-reporting patients.
*** Property crime-related refers to the type of violence that could not be categorised under rape/sexual, interpersonal violence, gang violence or unknown. Cases in this category included violence initiated to facilitate property theft from an individual.

Discussion

One of the key findings of a 2014 systematic review of the feasibility and effectiveness of community-level interventions involving emergency department data sharing to reduce alcohol-related violence, was that data sharing protocols can be cheaply and easily implemented into modern Emergency Department (ED) triage systems (17). There was general consensus among studies that this could be done with minimal cost, staff workload burden and impact to patient safety. Additionally, there was minimum risk to patient service and anonymity, risk of harm displacement to other licensed venues or increase to length of patient stay (17). The Cardiff Model is cost-effective with one study finding that for every $1 spent, nearly $15 in health system and over $19 in criminal justice system costs are saved (18).

To the best of our knowledge, this is the first study to attempt direct name matching between the health and the police to quantify the level of underreporting of interpersonal violence-related incidents. The matching method using personal identifiers suggest that only a small

For peer review only - http://bmjopen.bmj.com/site/about/guidelines.xhtml

fraction (approximately 15 %) of patients being treated for interpersonal violence at local health facilities will go on to report these as crimes to local police stations within a week. Conversely, only a small proportion of those who report a violent crime to the police go on to present to a health facility with an injury within a week.

The proportion of cases in this study of injuries arising from interpersonal violence which are not reported to the police (approximately 82%), is consistent with the findings of the Cardiff model which found that one half to two thirds of violence which results in hospital treatment is not known to the police (2). This finding is much lower when compared with other regions including in developed countries. According to a 2016 U.S. Department of Justice report, many crimes go unreported to law enforcement, including 53% of violent crime in 2015; 58% of simple assaults in 2015 and 43% of violent crime involving an injury in 2015 (19).

Possible reasons for the high level of underreporting (30%+ discrepancy between the level of under reporting in this study [82%) and the Cardiff model [50%]), can be found in the report of the 2012 Khayelitsha Commission of inquiry (also known as the O'Regan/Pikoli Commission) which investigated allegations of police inefficiency in Khayelitsha and the breakdown in relations between the Khayelitsha community and the police. Poor policing conditions characterised by police in the area being generally lower-ranked, poorly equipped, few in number, underqualified and reluctant to open cases, led to breakdown in trust with the community resulting in the lack of reporting (20). Other possible reasons for underreporting include the fear of reprisal from perpetrators, being turned away by police when presenting at the police station to report a crime, stigma/shame, or not perceiving an injury arising from interpersonal violence as a crime.

Type of injury

The majority of police reported crimes are related to assault with body part (push/kick/punch). Victims of knife crimes represented in the health data, which often cause worse injuries requiring medical intervention, are not reporting these incidents to police. Gang/Property crime-related episodes were more common among non-reporting patients, which may be due to a lack of confidence in the police's ability to protect them from reprisal, or if the victim perceived the gangs as being protected by or working with the police, in a known context of mistrust of police in the community of Khayelitsha (20). Additionally, the underreporting of knife wielding gang-related violence incidents in police statistics may be a contributing factor. This is supported by the lower proportion of younger male victims (age 16-24) reporting crime compared to those receiving treatment for an injury arising from interpersonal violence (31.2% vs 39.4%, P=0.017).

For peer review only - http://bmjopen.bmj.com/site/about/guidelines.xhtml

The scale in difference of sharp object injuries presenting at police (89 cases) and health facilities (474), means that 385 victims of violence treated for a sharp object injury at a health facility did not go on to report the crime to the police, which if added to the official police statistics would represent a 52% increase in all violent crimes reported. This provides evidence for an excess burden of injuries arising from interpersonal violence which is not being reported to police – a situation that is consistent with the premise of the Cardiff model.

Limitations

One of the limitations of this study is that approximately 10% of health records lacked names and were thus excluded from the matching process. A 2013 South African study in missing medical records found that up to 8% of hospital folders had the wrong names and that 17% of hospital folders had names without folder numbers (21).

For each one-week data collection period we should have extended the police data collection period to account for possible late reporting. Our results may therefore underestimate potential matching. In attempting to quantify the extent of this underreporting we considered the difference between time of presentation to the clinical facility and time recorded in the SAPS database and the percentage matched cases by day of week (Supplemental Table 1).

In most cases there was a modest difference between time of reporting (mean 7.9 hours, median 2.6 hours, 90th percentile 23 hours) and percent matching on the final day of the reporting period (22.7% on Wednesday) was higher than the average of 17.5%. we concluded that the extent of underreporting was negligible and would not materially affect our results.

No cases of poisoning were recorded in the health data. The current referral practice is for Michael M and Site B to refer their poisoning cases to KDH. At KDH most poisoning cases are triaged to the Medical department and thus these cases would not be recorded in the health data. The exception being cases of poisoning due to a corrosive substance such as bleach, which would be triaged to the Trauma/Surgical department due to the possible need for a surgical therapeutic intervention. It is a recommendation of study authors that the poisoning data from medical emergencies be included to inform this work going forward.

One of the weaknesses of this study is that patients who presented to a health facility but only lodged criminal cases later outside of the one-week study periods over the five years would not be included in the police dataset and therefore not be included in the matching process. Similarly, if victims of injuries arising from interpersonal violence presented to the police first

For peer review only - http://bmjopen.bmj.com/site/about/guidelines.xhtml

and did not present to the health facilities for treatment within the one-week study period, they were also not included in the matching process. The inclusion of these late reporting cases could decrease the overall proportion of interpersonal violence-related injuries that were reported to the police. Finally, patients experiencing inter-personal violence and who sought private health care or presented directly to the hospitals outside of the district (such as the tertiary referral hospital, Tygerberg Hospital) would not be included in the analyses. This may increase or decrease the estimate of concordance depending on whether these patients reported to the police.

Implications for policy and interventions

Murder and violence are often linked to alcohol with a 2016 analysis of murder dockets finding that in up to 48% of murders, the victim or perpetrator was intoxicated (22). Alcohol use was higher in patients who did not report violence than patients who did report violence to the police. This is of public health importance as alcohol has been identified as a major risk factor for and predictor of violence and is amenable to policy change to reduce alcohol harms. The current evidence base for measuring alcohol use within crime data will underestimate the extent of the problem because of the underreporting in routine crime statistics highlighted by this research.

Furthermore, with regard to local municipal alcohol policy on liquor outlet licences (23), reasons for limitation of liquor outlet licences may include, 'safety and social problems and/or concerns arising from alcohol abuse, alcohol related crimes, illegal criminal activity, etc, using data derived from the South African Police Service'. The local alcohol policy is heavily reliant on the SAPS data to guide license approvals, which this research has shown to be incomplete and flawed in its underestimation of interpersonal violence at the community level. Alcohol policy decisions made by Government at any level (local, district, provincial and national) that is informed by this data, may aggravate alcohol related harms.

The level of underreporting exposed by the merging of health and police datasets and matching exercise may speak to a larger question of police resourcing within Khayelitsha. The Khayelitsha commission of inquiry highlighted the insufficient police human resources within police stations in Khayelitsha as one of the reasons for community mistrust of the police(20). The current SAPS resource allocation model is based on the Theoretical Human Resource Requirement (THRR) and fixed establishment (FE) approach which was developed in 2012 (24) . The tool takes into consideration annual reported crime per police station (24) which suggests that current police

For peer review only - http://bmjopen.bmj.com/site/about/guidelines.xhtml

human resources deployed in Khayelitsha may be severely under resourced to deal with the 'real' burden of violence, when unreported health data is considered.

The validity of self-reported drinking using ED data from 16 countries reported findings suggesting that the overall validity of self-reported alcohol consumption before injury is high (88%) compared with a blood alcohol concentration (BAC) estimate obtained at the time of admission to the ED, with a caveat that demographic, drinking and injury characteristics may affect validity(25).

This study provides evidence showing that interpersonal violence at the community level cannot be estimated accurately relying solely on reported crimes to the police services. Injuries arising from interpersonal violence treated at health facilities must be included to more accurately define the level of interpersonal violence within a community.

Implementation of the Hospital Emergency Centre Tracking Information System (HECTIS), an electronic data collection system for Emergency Departments (ED), is currently underway at the Michael Mapongwana CHC and KDH. This will improve the monitoring of injuries arising from interpersonal violence (the majority of which are not reported to the police) and provide a method for the evaluation of violence prevention interventions. Additionally, this will provide the data infrastructure required for the potential implementation of the Cardiff model locally.

Preliminary findings from this research were shared with the Whole of Society Approach (WOSA) intergovernmental task team that operates in the community of Khayelitsha, with a mandate to reduce interpersonal violence. The full findings will be shared with the WOSA stakeholders as preliminary work to inform the full implementation of the Cardiff model in Khayelitsha early next year.

For peer review only

Conclusion

The matching exercise described above represents the first attempt within South Africa to match patients at health facilities treated for injuries arising from interpersonal violence with those of victims reporting violent crimes to police stations within the same community and time period. This matching study suggests that the majority of injuries arising from interpersonal violence presenting at health facilities in Khayelitsha are not reported to the police in Khayelitsha.

This study has broader implications regionally and nationally for the surveillance of injuries arising from interpersonal violence, for the police definition and surveillance of community

For peer review only - http://bmjopen.bmj.com/site/about/guidelines.xhtml

interpersonal violence, for community policing intelligence development (improving the configuration of violence heat maps on a real time basis) and finally for police resource utilisation and distribution, which should, in turn, impact positively on reducing crime and violence in the community, and reduce the burden on the Health services. The implementation of the Cardiff data sharing model should be adopted to inform violence reduction locally.

Ethics

Ethical permission to conduct this research was granted by the University of Cape Town Human Research Ethics Committee (UCT HREC 861/2016) and the South African Police Services Research Division (Application no. 3/34/2). With regard to the HST data, informed consent was obtained only from acute injury cases and where given, these individuals were interviewed by the data collectors, with only acute injury cases having the full data collection tool completed. Informed consent was granted by Groote Schuur Hospital for permission to access the Clinicom patient database. No minors were interviewed as part of this study.

There are no competing interests for any author.

AJ and RM developed the research proposal and were involved in the analysis and interpretation of the data. AC was involved with the analysis of the data including the matching protocol. LL and TO revised the work for content and approved the final version to be published.

There is no additional data available.

Funding

This work was supported by the International Development Research Centre (IDRC), Canada, and the UK Government's Department for International Development (DFID) (Grant number 107329–001) and the Western Cape Government Department of Health (Grant number WCDOH428/2013). The degree from which this study emanated was funded by the South African Medical Research Council (SAMRC) under the SAMRC Clinician Researcher (MD PhD) Scholarship Programme. RM is funded by the SAMRC. TO is supported by the National Institute for Health Research (NIHR) (16/137/34) using UK aid from the UK Government to support global health research. The views expressed in this publication are those of the authors and not necessarily those of the NIHR or the UK Department of Health and Social Care or SAMRC.

Figure 1. Participating health facilities and police stations in relation to study areas in the community of Khayelitsha

Figure 2. Case selection flow chart for health and police datasets

Figure 3. Personal identifier matching between datasets

For peer review only - http://bmjopen.bmj.com/site/about/guidelines.xhtml

For peer review only

References

1. CDC. Community Guidance for Violence Prevention Cardiff Model Toolkit [Internet]. Atlanta; 2018. Available from: https://www.cdc.gov/violenceprevention/fundedprograms/cardiffmodel/toolkit.html

2. Florence C, Shepherd J, Brennan I, Simon T. Effectiveness of anonymised information sharing and use in health service, police, and local government partnership for preventing violence related injury: experimental study and time series analysis. BMJ. 2011;342:d3313.

3. Group violence research. Cardiff Model for Violence Prevention Implementation of the Model in the UK International [Internet]. Vol. 44. Cardiff; 2018. Available from: https://static1.squarespace.com/static/57875c16197aea2902e3820e/t/5a03139f71c10ba51be806ee/1510151075166/vrg+cardiff+model+briefing+web.pdf

4. Kollar L, Sumner S, Bartholow B, Wu D, Moore J, Mays E, et al. Building capacity for injury prevention : a process evaluation of a replication of the Cardiff Violence Prevention Programme in the Southeastern USA. Inj Prev. 2019;1–8.

5. Boyle A a, Snelling K, White L, Ariel B, Ashelford L. External validation of the Cardiff model of information sharing to reduce community violence: natural experiment. Emerg Med J [Internet]. 2013;30(12):1020–3. Available from: http://www.ncbi.nlm.nih.gov/pubmed/23184923

6. Langton L, Berzofsky M, Krebs C, Smily-McDonald H. Victimizations not reported to police [Internet]. Bureau of Justice statistics. [cited 2017 Feb 17]. Available from: http://www.bjs.gov/content/pub/pdf/vnrp0610.pdf.2012

7. Cassidy T, Lloyd S, Bowman B, Myers J, Parry C, Makanga T, et al. Authors : [Internet]. Cape Town; 2013. Available from: https://open.uct.ac.za/bitstream/handle/11427/28271/Cassidy_Survey violence alcohol.pdf?sequence=3

8. Government WC. Western Cape Safety Plan Working Document. Cape Town; 2019.

9. City of Cape Town. City of Cape Town – 2011 Census Suburb Khayelitsha [Internet]. Cape Town; 2011. Available from: https://www.capetown.gov.za/en/stats/2011CensusSuburbs/2011_Census_CT_Suburb_Khayelitsha_Profile.pdf

10. Barolsky V. Violence in Khayelitsha: finding a way out. Cape Town; 2014.

11. Mureithi L, Africa A, Wandai M, Schaik N Van, Naledi T, Matzopoulos R, et al. Injury Morbidity Surveillance in Nyanga and Khayelitsha in the Western Cape. Vol. 27. Cape Town; 2016.

12. Government WC. New Patient Administrative (AR) and Billing system at Prince Albert Hospital [Internet]. Western Cape Government news. 2020 [cited 2020 Nov 17]. Available from: https://www.westerncape.gov.za/news/new-patient-administrative-ar-and-billing-system-prince-albert-hospital

13. SAPS. SAPS crime defintions. SAPS Website. 2019.

14. R Core Team. R: A language and environment for statistical computing [Internet]. R Foundation for Statistical Computing. 2013 [cited 2020 Sep 3]. Available from: http://www.r-project.org/

15. Zeileis A. Object-Oriented Computation of Sandwich Estimators. J Stat Softw. 2006;16(9):1–16.

16. Doan A, Halevy A, Ives Z. Principles of Data Integration. 1st ed. Morgan Kaufmann; 2012. 95–119 p.

For peer review only - http://bmjopen.bmj.com/site/about/guidelines.xhtml

17. Droste N, Miller P, Baker T. Review article: Emergency department data sharing to reduce alcohol-related violence: A systematic review of the feasibility and effectiveness of community-level interventions. Emerg Med Australas [Internet]. 2014;26(4):326–35. Available from: http://doi.wiley.com/10.1111/1742-6723.12247

18. Holder, Yvette et al. Injury Surveillance Guidelines. Geneva; 2001.

19. Morgan R, Kena G. Criminal Victimization,2016:Revised [Internet]. Bureau of Justice Statistics. 2018. Available from: https://www.bjs.gov/content/pub/pdf/cv16.pdf

20. WCG. Report of the Commission of Inquiry into Allegations of Police Inefficiency and a Breakdown in Relations between SAPS and the Community of Khayelitsha. Cape Town; 2014.

21. Wegner L, Rhoda A. Missing medical records : an obstacle to archival survey-research in a rural community in South Africa. South African J Physiother. 2013;15–9.

22. SAPS. Crime statistics South Africa [Internet]. Cape Town; 2016. Available from: https://www.saps.gov.za/services/crimestats.php

23. Municipality D. Drakenstein Municipality Liquor Outlet and Tavern draft Policy [Internet]. Cape Town: Drakenstein Municipality; 2020. Available from: http://www.drakenstein.gov.za/docs/Documents/DM Liquor Policy - 3rd draft_20200723.pdf

24. Government WC. Analyses of Human Resource Allocation for South African Police Service with specific reference to the Western Cape. Cape Town; 2017.

25. Cherpitel C, Ye Y, Bond J, G B, Macdonald S, Stockwell T, et al. Validity of self-reported drinking before injury compared with a physiological measure: cross national analysis of Emergency department data from 16 countries. 2006.

For peer review only

For peer review only - http://bmjopen.bmj.com/site/about/guidelines.xhtml

Figure 1

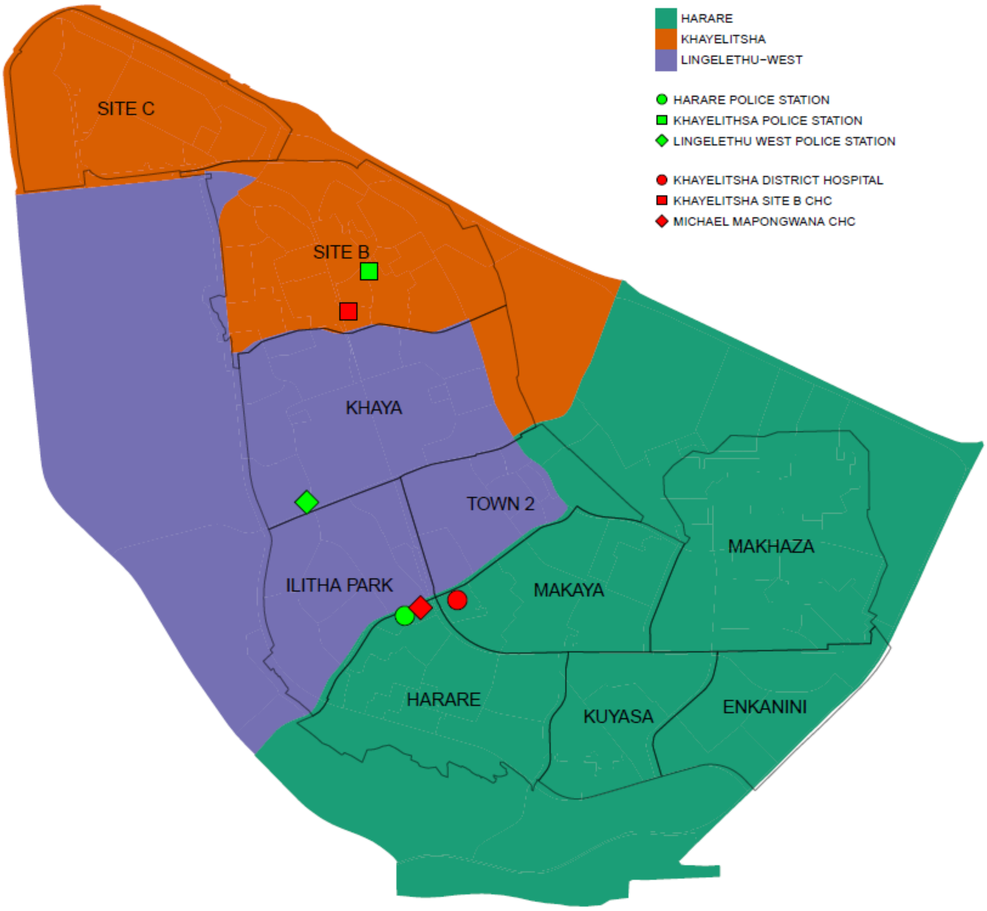

Figure 1. Participating health facilities and police stations in relation to study areas in the community of Khayelitsha

ew only

Figure 2

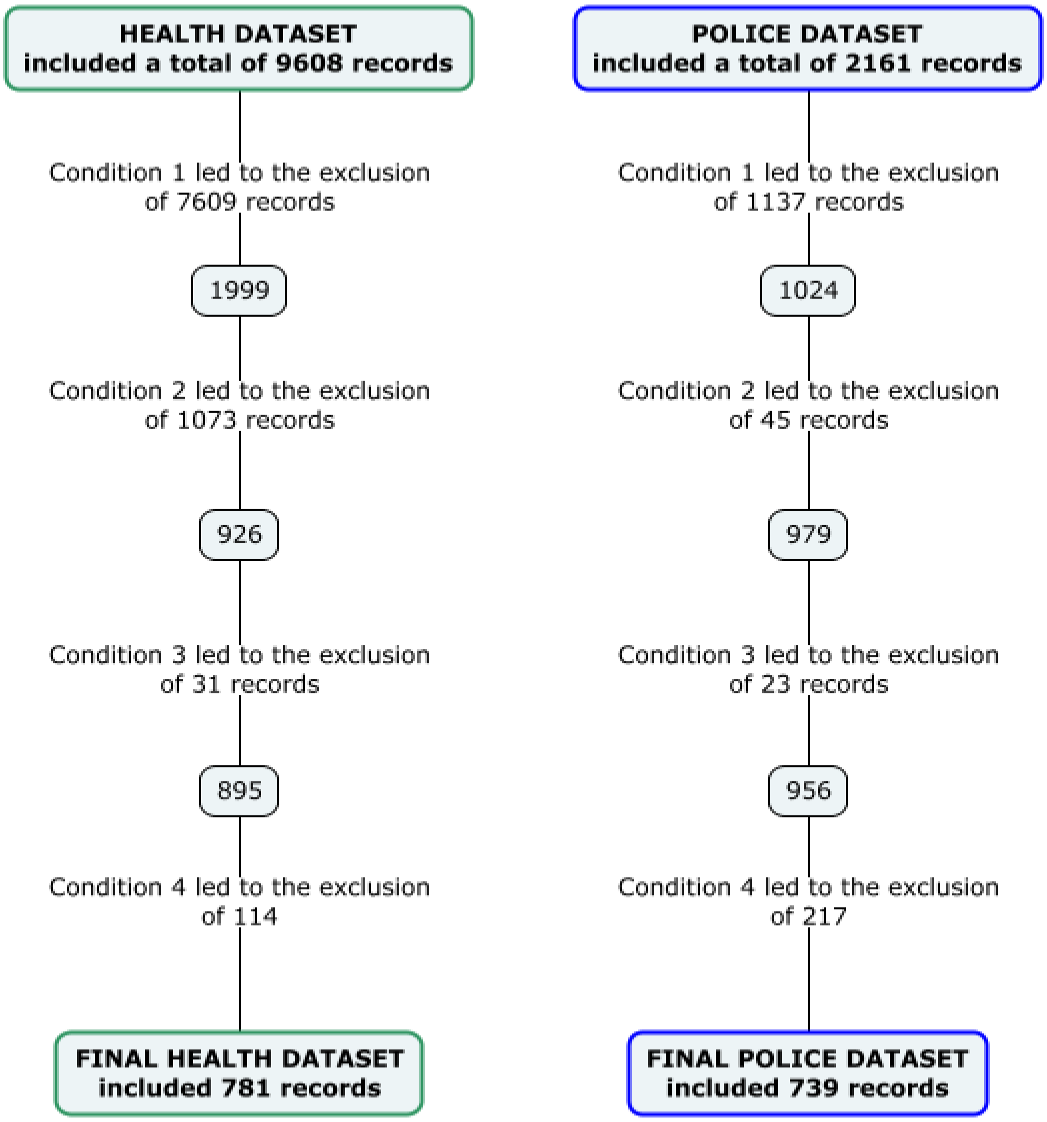

KEY:

Health condition 1: Cases that were excluded from the health data included medical cases not related to injuries and injuries not caused by violence perpetrated on the victim by another subject

Police condition 1: Cases that were removed from the police data include cases other than the types of crimes described in variables section

Condition 2: events that occurred outside the ten areas of Khayelitsha

Condition 3: cases in which the victim was younger than 16 years

Condition 4: events that occurred outside the time period

Figure 2. Case selection flow chart for health and police datasets

Figure 3

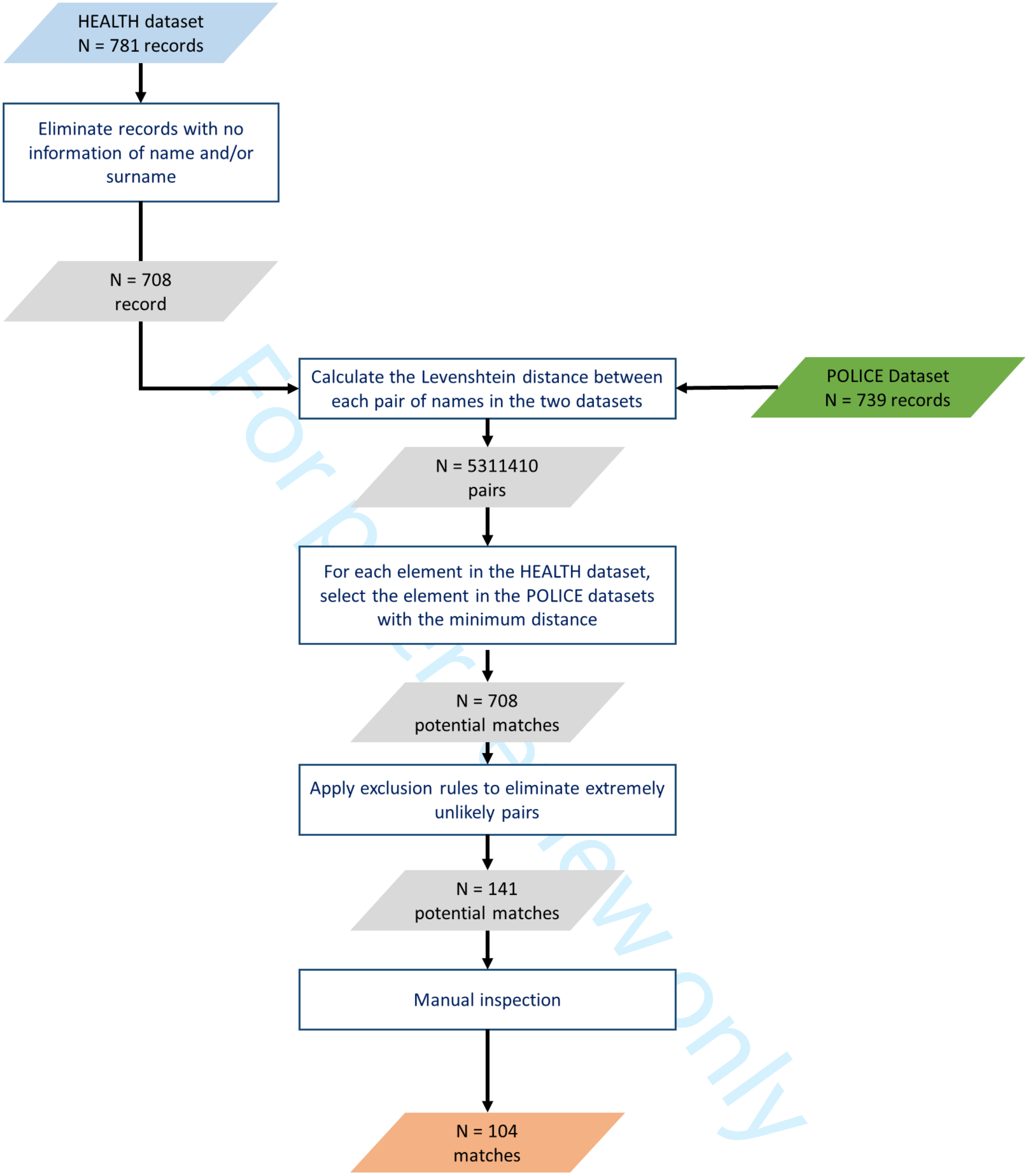

Figure 3. Personal identifier matching between datasets

Supplemental Table 1. Matching proportion per day of week of presentation to the clinical facility

DAY	MATCHED	TOTAL	PERCENT (%)
Monday	14	89	15,7
Tuesday	12	47	25,5
Wednesday	10	44	22,7
Thursday	5	37	13,5
Friday	15	70	21,4
Saturday	27	182	14,8
Sunday	21	239	8,8

For peer review only
